# Deletion of metal transporter *Zip14* (*Slc39a14*) produces skeletal muscle wasting, endotoxemia, Mef2c activation and induction of miR-675 and Hspb7

**DOI:** 10.1038/s41598-020-61059-2

**Published:** 2020-03-04

**Authors:** Jinhee Kim, Tolunay Beker Aydemir, Felix R. Jimenez-Rondan, Courtney H. Ruggiero, Min-Hyun Kim, Robert J. Cousins

**Affiliations:** 1 Food Science and Human Nutrition Department, Center for Nutritional Sciences, College of Agricultural and Life Sciences, University of Florida, Gainesville, FL 32611 USA; 2 Rutgers Medical School, Newark, NJ USA; 3 Cornell University, Ithaca, NY USA; 4 University of Michigan, Ann Arbor, MI USA

**Keywords:** Metabolism, Metabolic syndrome, Medical research

## Abstract

Skeletal muscle represents the largest pool of body zinc, however, little is known about muscle zinc homeostasis or muscle-specific zinc functions. *Zip14* (Slc39a14) was the most highly expressed zinc transporter in skeletal muscle of mice in response to LPS-induced inflammation. We compared metabolic parameters of skeletal muscle from global *Zip14* knockout (KO) and wild-type mice (WT). At basal steady state *Zip14* KO mice exhibited a phenotype that included muscle wasting and metabolic endotoxemia. Microarray and qPCR analysis of gastrocnemius muscle RNA revealed that ablation of *Zip14* produced increased muscle *p-Mef2c*, *Hspb7* and *miR-675-5p* expression and increased p38 activation. ChIP assays showed enhanced binding of NF-$\kappa \beta$ to the *Mef2c* promoter. In contrast, LPS-induced systemic inflammation enhanced Zip14-dependent zinc uptake by muscle, increased expression of Atrogin1 and MuRF1 and markedly reduced *MyoD*. These signatures of muscle atrophy and cachexia were not influenced by *Zip14* ablation, however. LPS-induced *miR-675-3p* and *-5p* expression was Zip14-dependent. Collectively, these results with an integrative model are consistent with a Zip14 function in skeletal muscle at steady state that supports myogenesis through suppression of metabolic endotoxemia and that *Zip14* ablation coincides with sustained activity of phosphorylated components of signaling pathways including p-Mef2c, which causes Hspb7-dependent muscle wasting.

## Introduction

Skeletal muscle represents the body compartment in mammals with the largest proportion of total body zinc. For humans, percentage estimates average 57% of total body zinc^1^. Despite the magnitude of zinc abundance in this organ, little is known regarding the physiologic roles of zinc in skeletal muscle or the transport mechanisms that maintain zinc homeostasis for functions in skeletal muscle.

At an integrative level, skeletal muscle is viewed as a responder to stressors such as endotoxin and pro-inflammatory cytokines^2^. In congruence with that role is the expression of toll-like receptor 4 (TLR4) in skeletal muscle^3^. Specifically, muscle TLR4 is activated by endotoxin (lipopolysaccharide; LPS) and causes altered substrate metabolism, including enhanced glucose utilization and decreased fatty acid oxidation. Skeletal muscle TLR4 has been proposed as a link between metabolic endotoxemia and some metabolic disorders^4,5^. Muscle loss is a known consequence of metabolic endotoxemia^6^.

Our interest in control of zinc metabolism has focused on the physiologic regulation of zinc transporter expression. We conducted a screen of transcripts for all 24 of the ZnT and Zip family of zinc transporters using quantitative PCR (qPCR) in numerous tissues of mice following treatment with LPS^7^. Of the 24 transcripts studied, *Zip14* mRNA was the most highly up-regulated following LPS treatment in two tissues, i.e. white adipose tissue (WAT) and skeletal muscle. Specifically, in WAT *Zip14* ablation produced hypertrophic adiposity and increased circulating leptin levels coincident with increased activation of NF-$\kappa \beta$ and STAT3 pathways^8^.

Based upon the recognized responsiveness of skeletal muscle to endotoxins and downstream metabolic events that occur as a result^3,9,10^, we explored the phenotypic consequences of whole-body *Zip14* ablation (*Zip14* KO) in skeletal muscle. We report here that *Zip14* KO mice have muscle wasting as measured by physical and biochemical indices that are concurrent with inflammatory signatures.

## Results

### Acute endotoxemia induced by LPS increases ZIP14 expression in skeletal muscle

**Figure 1:**
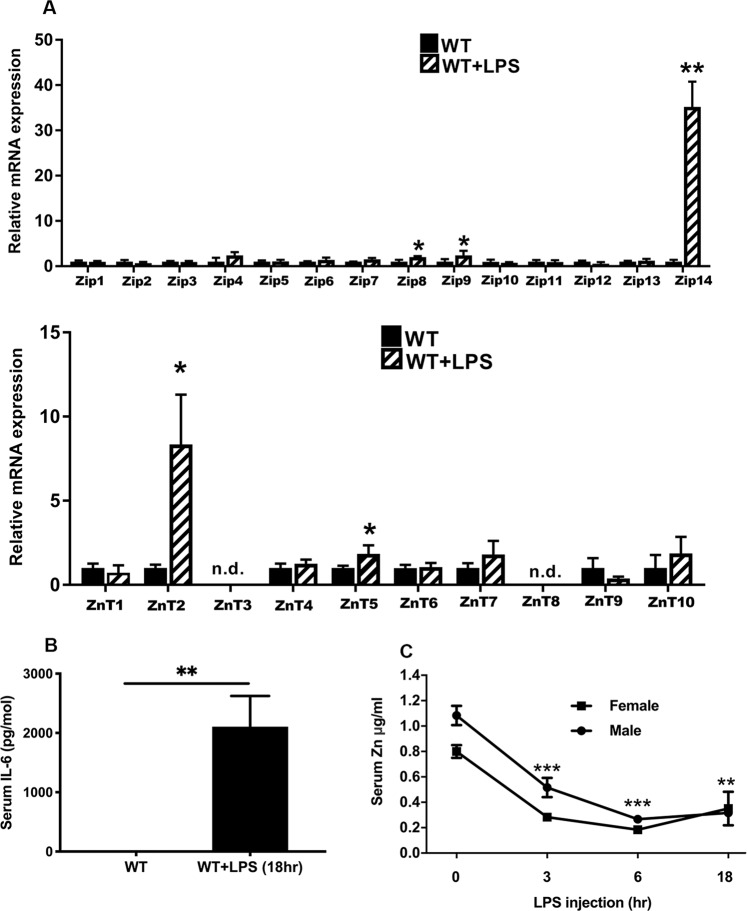
Expression of Zip and ZnT transcripts in skeletal muscle during acute inflammation induced with lipopolysaccharide (endotoxin). (**A**) Relative abundance of Zip mRNAs and ZnT mRNAs in gastrocnemius muscle of WT mice at 18 h after LPS. (**B**) Serum IL-6 concentrations at 18 h after LPS. (**C**) Serum zinc concentrations of male and female mice at 0–18 h after LPS. The LPS dose was 2 mg/kg (i.p.). Values are means $\pm$ SEM, n = 3–4 mice per treatment group. *P < 0.05; **P < 0.01; ***P < 0.001. Solid bars are WT mice; shaded bars are LPS-treated WT mice. nd = not detectable.

We hypothesized that zinc transport in skeletal muscle would be increased during inflammation through increased ZIP14, since expression of this metal transporter is increased in liver^11^ and adipose tissue^8^ following endotoxin (LPS) treatment and in liver during sepsis^12^. To establish which transporters might be responsive to inflammation in skeletal muscle, a screen of all *ZnT* and *Zip* transcripts was conducted using individual qPCR assays with RNA isolated from gastrocnemius muscle (GM) tissue from female WT mice following LPS administration. After LPS major increases in relative abundance of *Zip14* and *ZnT2* mRNAs was demonstrated with fold changes (FC) of +25 and +8, respectively (Fig. 1A). Significant changes (P < 0.05) of much lower magnitude were found for *Znt5*, *Zip7* and *Zip8* mRNAs following LPS. An acute inflammatory response was confirmed through the increase in serum IL-6 concentrations (Fig. 1B) and the hypozincemia produced by LPS treatment (Fig. 1C).

### Zip14 knockout mice exhibit altered zinc metabolism in gastrocnemius muscle and metabolic endotoxemia

**Figure 2:**
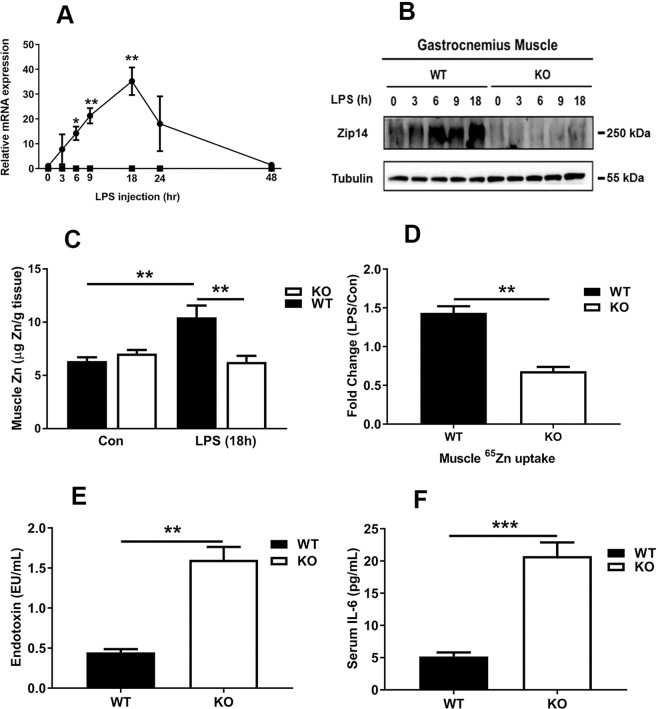
Metabolic endotoxemia at steady state and Influence of acute inflammation induced by LPS on skeletal muscle of wild type and *Zip14* knockout mice. (**A**) Induction of *Zip14* mRNA, 3–48 h after LPS. (**B**) Western analysis of induction of muscle Zip14 protein 0–18 h after LPS. Each lane is pooled sample from n = 4 per group. Blots were cut horizontally at the appropriate molecular mass and incubated with the appropriate antibody for the target protein and show contiguous lanes. The blots are representative of multiple experiments. (**C**) Muscle Zn concentration in WT and *Zip14* KO mice 18 h after LPS. The LPS dose was 2 mg/kg (i.p.). (**D**) Uptake of orally administered 65Zn into muscle in WT and *Zip14* KO mice. (**E**) Serum endotoxin levels. (**F**) Serum IL-6 concentrations. Values are means $\pm$ SEM, n = 4. *P < 0.05; **P < 0.01; ***P < 0.001.

To further characterize the influence of acute inflammation on ZIP14 expression in muscle, we compared the kinetics of Zip14 induction and parameters of zinc metabolism in WT mice to those in *Zip14* KO mice following LPS. *Zip14* transcripts peaked at 18h after LPS in the WT mice, but were not changed in the KO mice (Fig. 2A). Compared to *TATA binding protein* mRNA and *18S* RNA, *glyceraldehyde-3-phosphate dehydrogenase* (*Gapdh*) mRNA gave the most constant abundance level over the 18h time course and was used for normalization in these time course assays. Western analysis revealed Zip14 protein abundance increased in GM after LPS administration in the WT mice (Fig. 2B).

Total zinc concentrations increased to about 5 $\mu$g/g of GM tissue of the WT mice by 18h after LPS (Fig. 2C). These results suggest that *Zip14* ablation prevented the LPS-stimulated increase in zinc transport into muscle. In support of that suggestion, when ${}^{{\rm{65}}}$Zn was given to the mice as an oral dose, the ${}^{{\rm{65}}}$Zn taken up by the GM of the KO mice was about 50$\%$ of the ${}^{{\rm{65}}}$Zn taken up by the GM of the WT controls (Fig. 2D). By contrast, manganese, non-heme iron (NHI) and total phosphorus concentrations of the GM were not influenced by LPS (Supplementary Fig. 1). The zinc content of the soleus muscle was not influenced by LPS administration in mice of either sex (Supplementary Fig. 1). This difference could reflect the glycolytic properties of the GM. The responsiveness of GM from female mice to LPS at this dose level in previous experiments^7^, and as shown here, led to the decision to use females for subsequent experiments except the morphological comparisons.

Serum endotoxin levels were increased in the KO mice compared to the WT mice indicating a loss of some intestinal barrier function due to *Zip14* ablation (Fig. 2E), which is in agreement with previous observations^7,13^. Similarly, at steady state, serum IL-6 concentrations were increased significantly in the KO mice (Fig. 2F). Increased circulating levels of both endotoxin and IL-6 are signatures of mild inflammation described as metabolic endotoxemia.

### Zip14 ablation produces structural defects in skeletal muscle at basal steady state

**Figure 3:**
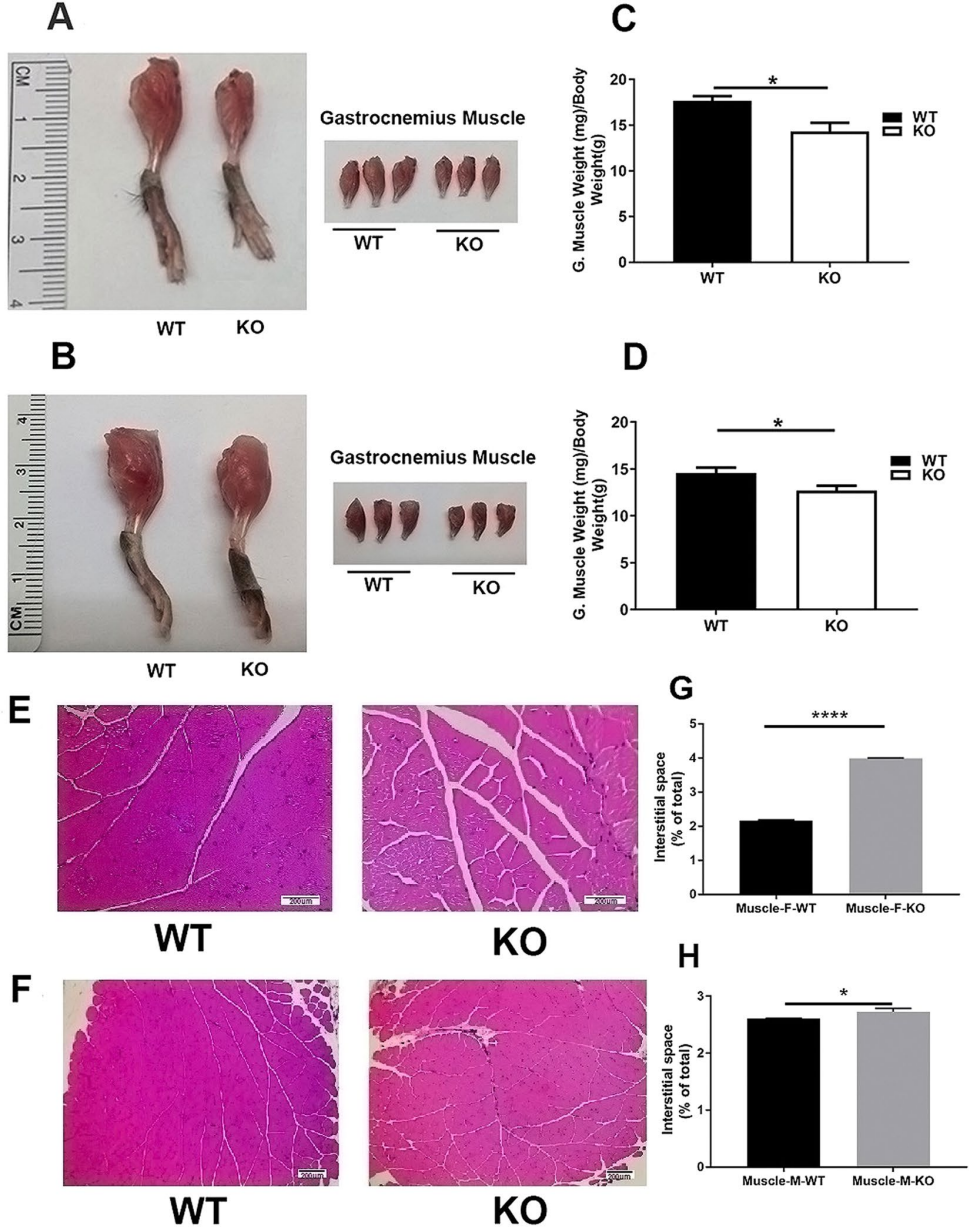
*Zip14* knockout mice exhibit skeletal muscle wasting at steady state. Representative images of hind legs of WT and *Zip14* KO female (**A**) and male (**B**) mice showing reduced length and mass and representative excised gastrocnemius muscle from both genotypes. The mice used were 12–16 wks of age. Ratio of muscle weight to body weight for female (**C**) and male (**D**) mice of both genotypes. H$\&$E stained sections of these muscles from female (**E**) and male (**F**) mice. Magnification is 40x (**E**) and 20x (**F**), respectively. Interstitial area of muscle from the H$\&$E stained images (**G**, female mice; **H**, male mice; n = 3) Numerical data are means $\pm$ SEM. *P < 0.05, ****P < 0.0001.

Morphology of the leg musculature of the hind limbs was influenced by *Zip14* ablation. The length and apparent muscle mass were less in the female KO mice (Fig. 3A). Somewhat less pronounced differences in muscle wasting were found in KO male mice (Fig. 3B). Ratios of GM weight to body weight were significantly less in both female and male KO mice (P < 0.05; Fig. 3C,D). Organization of GM structure was evaluated using H$\&$E stained sections at 20x or 40x magnification (Fig. 3E female muscle at 40x and Fig. 3F male muscle at 20x). Muscle from both female and male KO had greater (P < 0.0001 and P < 0.05, respectively) interstitial space between bundles from muscle fibers than muscle from WT mice (female mice, Fig. 3G; male mice, Fig. 3H). Gel electrophoresis of GM lysate followed by Coomassie staining revealed no obvious differences in muscle proteins between the KO and WT mice (Supplementary Fig. 2).

### Genes that influence muscle wasting are differentially expressed in gastrocnemius muscle of Zip14 knockout mice at steady state

**Figure 4:**
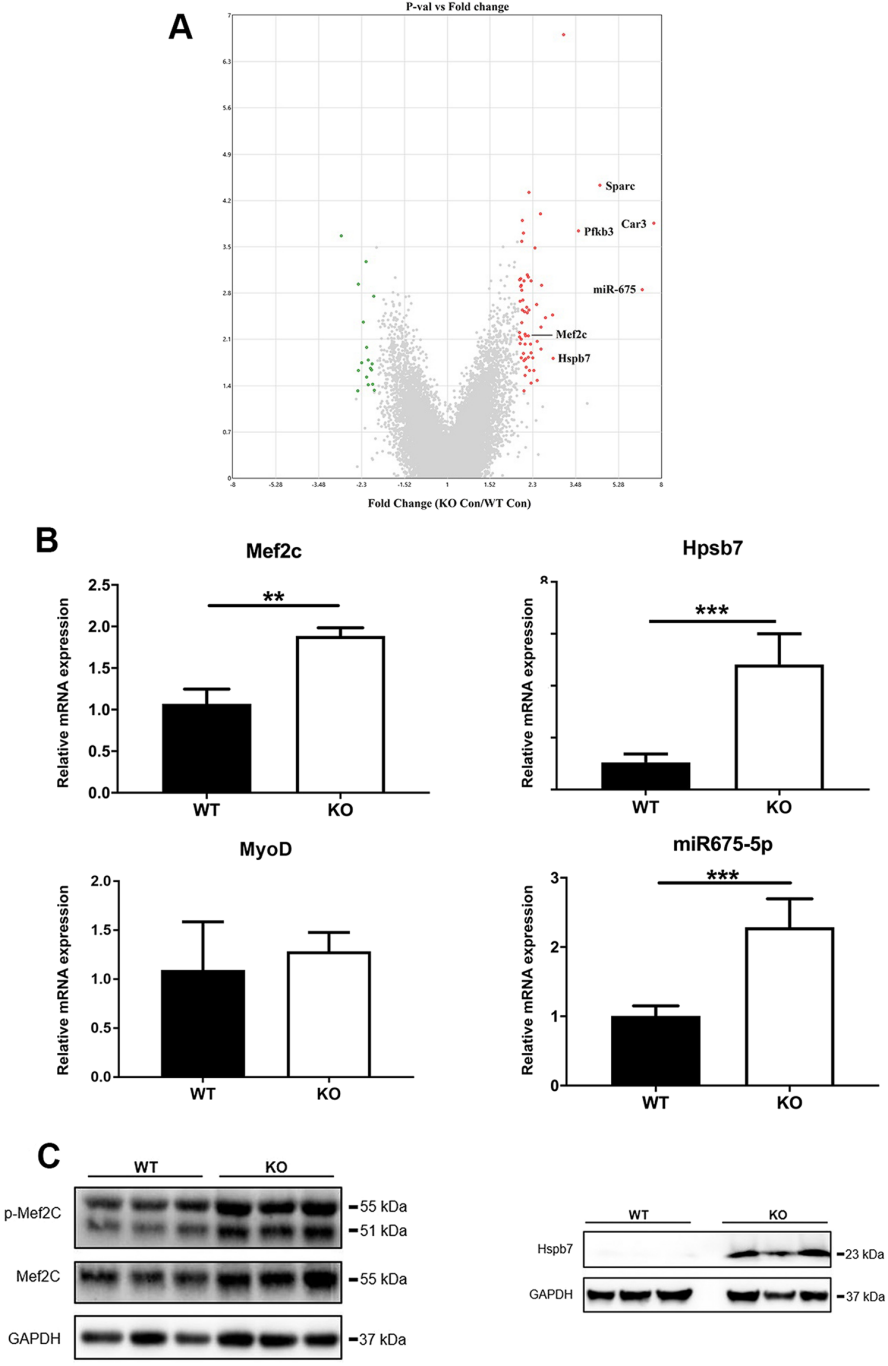
Genome-wide transcriptome comparisons using microarrays identify differentially expressed genes in skeletal muscle of wild type and *Zip14* KO mice at steady state. (**A**) Volcano plot of differentially expressed genes. Total RNA was isolated from individual gastrocnemius muscle tissue of WT and *Zip14* knockout mice and microarray analysis was performed. Treatment groups had n = 3–4 mice. A total of 76 transcripts were designated as differentially expressed between the genotypes at a FC $\ge$ +2 or $\le \,-\,2$. Data are FC vs. probability expressed as a ratio of KO/WT. (**B**) Relative mRNA levels for *Mef2c*, *Hspb7*, *MyoD* and *miR-675-5p* as subsequently measured by qPCR. Values are means $\pm$ SEM, n = 4 mice per group. **P < 0.01; ***P < 0.001. (**C**) Western analysis of Hsbp7 and Mef2c protein levels. Each lane represents a lysate from one mouse. Blots were cut horizontally at the appropriate molecular mass and incubated with the appropriate antibody for the target protein and show contiguous lanes. The blots are representative of multiple experiments.

Microarray analysis of total RNA from GM was used to compare transcript abundance to identify genes that were differentially expressed with *Zip14* ablation. Ranking of transcripts for 22,206 annotated genes on the microarray revealed 76 differentially expressed genes at a FC of $\ge$+2.0 or $\le \,-\,2.0$ between the WT and KO mice at basal steady state (Supplementary Table 1). FCs vs. probability values for those transcripts, expressed as a ratio of KO/WT, is shown in a volcano plot (Fig. 4A). *Carbonic anhydrase 3* (*Car3*) mRNA was the most up-regulated (Table 1). The next was *H19* RNA, a long non-coding RNA (*lncRNA*), which generates the microRNAs, *miR-675-3p* and *miR-675-5p*^14,15^. Transcript levels for *secreted acidic cysteine-rich glycoprotein* (*Sparc*), also called osteonectin, and *6- phosphofructo-2 kinase/fructose-2,6-bisphosphatase 3* (*Pfkfb3*) had high abundances. *Heat shock protein family member 7* (*Hspb7*) and *decorin* (*Dcn*) had FCs of +2.8 and +2.6, respectively. Of great relevance to muscle integrity is the up-regulation of *Mef2c* (FC +2.2) in muscle of the *Zip14* KO mice. A total of 17 genes were down-regulated (FC <$\,-2.0$) in *Zip14* KO mice compared to WT mice. None were obviously connected to muscle-specific functions (Supplementary Table 1). Separately, qPCR assays were used to confirm that *Mef2c*, *Hspb7* and *miR675-5p* mRNAs were up-regulated in the GM of the KO mice (Fig. 4B). Expression of *MyoD* (Fig. 4B) and *Cdc6* were not different between the genotypes, while *miR-675-3p* was decreased (Supplementary Fig. 3A,B). Western analysis of GM lysates confirmed that Mef2c and Hspb7 proteins were increased in abundance in GM of the *Zip14* KO mice (Fig. 4C). Since both proteins are essential for skeletal muscle integrity^16–19^, their increased expression most likely contributes to the muscle wasting phenotype found with *Zip14* ablation in mice at steady state.

**Table 1:** Selected up-regulated genes in gastrocnemius muscle of Zip14 KO mice at steady state. Complete list of 76 deferentially expressed at FC $\ge$ +2 or $\le \,-\,2$ is in Supplementary Table 1.

Gene Name	Fold Change	Probability
Carbonic anhydrase 3	7.41	0.0001
H19/miR-675	6.63	0.0014
Secreted acidic cysteine rich glycoprotein	4.41	3.69E-05
6-Phosphofructo-2-kinase/fructose-2,6-biphosphatase 3	3.58	0.0002
Heat shock protein family, member 7	2.8	0.0154
Decorin	2.63	0.0038
Myocyte enhancer factor 2c (Mef2c)	2.2	0.007

**Figure 5:**
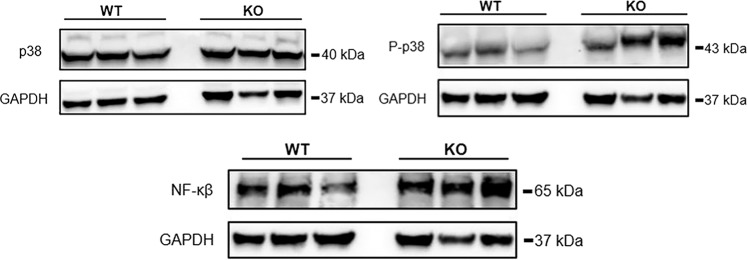
Comparison of specific pathway activation in skeletal muscle of wild type and *Zip14* KO mice at steady state. Western analysis of activation of p38, p-p38 and NF-$\kappa \beta$. Each lane represents a lysate from one mouse. Blots were cut horizontally at the appropriate molecular mass and incubated with the appropriate antibody for the target protein and show contiguous lanes. The blots are representative of multiple experiments.

Dysregulated pathways that influence phosphorylation could contribute to the biochemical mechanism of the muscle wasting phenotype found with *Zip14* ablation. p38 mitogen-activated protein kinase (p38), when activated by LPS leading to increased phosphorylation^20^, has been shown to phosphorylate transcription factors including Mef2^21^. While p38 protein levels were similar and p-p38 levels were increased this likely suggests *Zip14* influences phosphorylation of this inflammation-related transcription factor (Fig. 5A). Furthermore, at steady state NF-$\kappa \beta$ is activated to a greater extent in muscle of the KO genotype suggesting that transcription of some inflammation-related genes is enhanced at steady state. A wasting phenotype is also supported by the up-regulation of *Tgf*$\beta$ mRNA in the KO mice (Supplementary Fig. 3C).

### ChIP analysis of the Hp and Mef2c promoters

**Figure 6:**
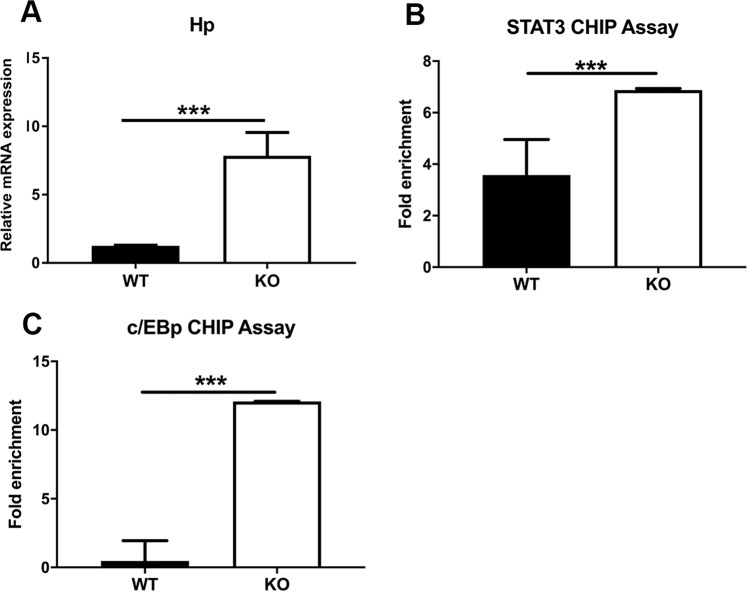
Elevation of haptoglobin expression in skeletal muscle as influenced by *Zip14* ablation in mice at steady state. (**A**) Abundance of *Hp* mRNA from the individual qPCR analysis. ChIP analysis of the *Hp* promoter performed with DNA from muscle using antibodies for (**B**) STAT3 and (**C**) C/EBP. Values are means $\pm$ SEM, n = 4 mice per group. ***P < 0.001.

The differential expression of specific genes in the *Zip14* KO genotype at steady state and after LPS led us to compare two examples of inflammation-responsive genes as clues to understand the relationship between Zip14, their expression and muscle wasting. First, we chose *Haptoglobin* (*Hp*) because it is a sentinel gene for inflammation and the microarray data showed expression was up-regulated to a greater extent in muscle of *Zip14* KO mice than WT mice (Supplementary Tables 2 and 3). We confirmed the microarray analysis using qPCR with *Hp* mRNA levels being greater in the GM of the KO mice at steady state and markedly increased following LPS administration (Fig. 6A). The murine *Hp* promoter has one STAT3 and two C/EBP elements within the first 1kb^22^. Our ChIP assays demonstrate that both transcription factors bound to a greater extent to *Hp* promoter DNA from the KO mice at steady state and in rough proportion to the abundance of their binding sites (Fig. 6B,C). This suggests that at steady state, inflammation-related signaling pathways leading to STAT3- and/or C/EBP- mediated transcription are more activated in muscle of the *Zip14* KO mice.

**Figure 7:**
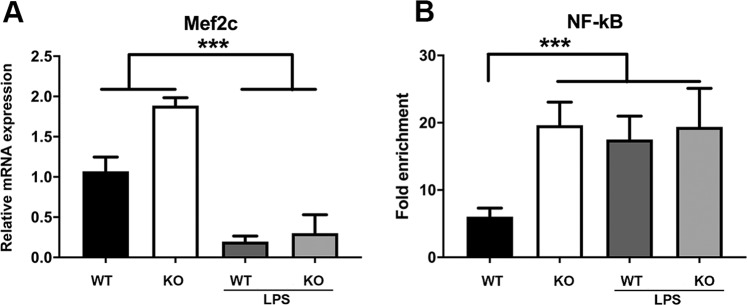
Comparison of *Mef2c* expression and promoter activation in skeletal muscle from wild type and *Zip14* KO mice at steady state and during acute inflammation as induced by LPS. (**A**) Levels of *Mef2c* mRNA as measured by qPCR. (**B**) ChIP analysis of *Mef2c* promoter binding of NF-$\kappa \beta$ using muscle DNA. Values are means $\pm$ SEM, n = 4 per genotype. ***P < 0.001. The LPS dose was 2 mg/kg (i.p.) 18 h before sacrifice.

Since *Hspb7* is regulated by Mef2 transcription factors^23^, and the Mef2c promoter has a NF-$\kappa \beta$ binding site^24^, we reasoned that the increased Mef2c expression in GM of the *Zip14* KO mice (Fig. 4A–C and 7A) could be due in part to increased NF-$\kappa \beta$ stimulated transcription. Indeed, a three-fold increase in NF-$\kappa \beta$ binding to the *Mef2c* promoter was observed with DNA isolated from the GM of the *Zip14* KO mice indicating enhanced transcriptional activation (Fig. 7B). This finding suggests that up-regulation of Hspb7 is mediated by Mef2c acting in response to the enhanced systemic proinflammatory activity of the *Zip14* KO mice. The lack of further increases in NF-$\kappa \beta$ binding in muscle of LPS-treated mice may indicate that over-riding repressive factors prevail to repress further Mef2c expression in acute systemic inflammation. Of note is that such LPS-induced responses are independent of Zip14 expression.

### Transcriptional responses of atrophy signatures to LPS-induced acute systemic endotoxemia in skeletal muscle are independent of ZIP14 expression

We screened the changes in mRNA expression that occur as the result of *Zip14* ablation in GM after LPS-induced acute inflammation using microarrays. Comparisons of $\pm$ LPS administration revealed that from the total of 22,206 annotated genes on the microarray, 397 and 522 were differentially expressed in WT vs *Zip14* KO mice, respectively, based on ranking by FC of $\ge$+2.0 or $\le \,-\,2.0$ (Supplementary Tables 2 and 3). Hierarchical clustering of those RNAs from individual mice is shown in a heat map (Supplementary Fig. 4). Metallothionein 1 (*Mt1*), metallothionein 2 (*Mt2*) and lipocalin 2 (*Lcn2*) served as sentinel markers of induction of acute systemic endotoxemia in response to LPS administration.

**Figure 8:**
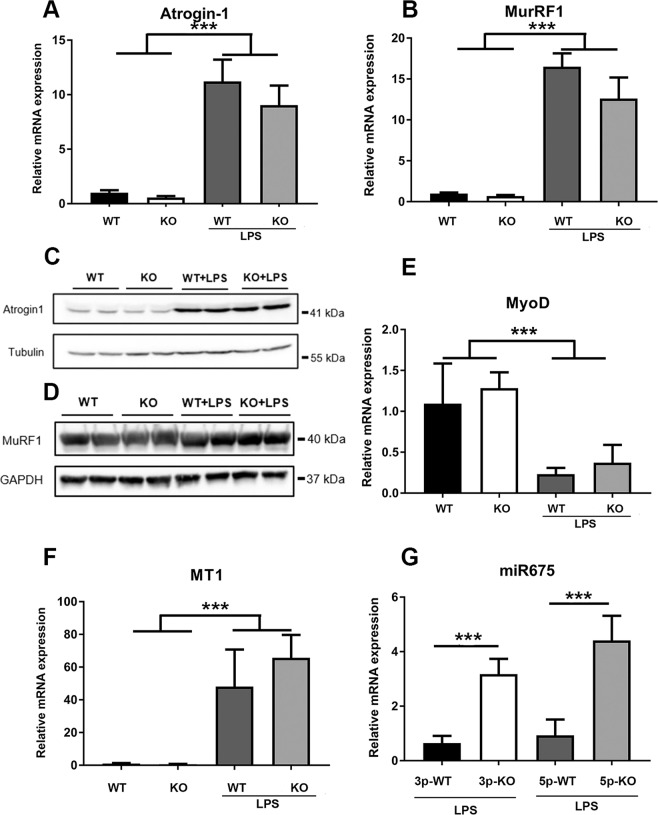
Transcript abundance of *Atrogin1*, *MuRF1*, *MyoD*, *Metallothionein 1*, *miR-675-3p*, and *miR-675-5p* and Atrogin1 and MuRF1 protein in skeletal muscle as influenced by *Zip14* ablation and acute inflammation induced by LPS. (**A**, **B**, **E**–**G**) Relative transcript abundances as measured by qPCR. (**C**,**D**) Relative protein abundance was measured by western analysis. Each lane represents a lysate from one mouse. The LPS dose was 2 mg/kg (i.p.) given 18 h before sacrifice. Values for mRNA levels are means $\pm$ SEM, n = 4 mice per group. ***P < 0.001.

qPCR assays confirmed that transcripts specifically related to skeletal muscle atrophy including *Atrogin1*, also referred to as *Fbxo32* or *MAFbx*, and *MuRF1* (muscle-specific ring finger protein 1), also referred to as *Trim63*, were highly induced by LPS (Fig. 8A,B). Importantly, the robust increase in expression of these genes in response to LPS treatment was comparable in mice with *Zip14* ablation and WT mice. Western analyses show that both proteins are increased in skeletal muscle of the LPS-treated mice, but of particular note, the increase is comparable in both WT and *Zip14* KO mice (Fig. 8C,D). *MyoD* expression was markedly depressed by LPS treatment, but the repression, as with *Murf1* and *Atrogin1* induction, was independent of Zip14 expression (Fig. 8E). Expression of *Mt1* in GM is shown for comparison (Fig. 8F). Of considerable interest is the marked increase in expression of both *miR675-3p* and *miR-675-5p* in muscle from the Zip14 KO mice after LPS administration (Fig. 8G).

## Discussion

The experiments described here were designed to establish if ZIP14 deletion would influence muscle metabolism and function. Specifically, we have shown that Zip14 expression in the glycolytic gastrocnemius muscle, compared to other known zinc transporters, is uniquely stimulated by acute administration of LPS and the increased zinc accumulation that follows is Zip14-dependent. At steady state, global *Zip14* ablation creates systemic metabolic endotoxemia that is concurrent with numerous phenotypic changes including skeletal muscle wasting. These acute (LPS-induced) vs. low level chronic states of endotoxemia are known to produce high and low toll-like receptor 4 (TLR4) activation in skeletal muscle, respectively^3^. Here we have compared responses of wild type and *Zip14* global KO mice using these two levels of endotoxemia.

Previously, our studies of the zinc transporter Zip14 (Slc39a14), have demonstrated in mice that Zip14, through its zinc transporting function, is able to influence inflammation in adipose tissue, control the regenerative capacity of the liver and facilitate adaptation to hepatic endoplasmic reticulum stress^8,25,26^. Furthermore, global ablation of *Zip14* results in a metabolic endotoxemia, a low grade inflammation, with characteristic elevated plasma endotoxin levels^7,13^. The likely cause of the increase in plasma endotoxin is that, without Zip14, zinc was trapped within vesicles in enterocytes, thus restricting cytosolic zinc to levels that are insufficient for maintenance of tight junction assembly and proper intestinal barrier function^13^. Another salient feature of the murine *Zip14* KO phenotype was elevated serum IL-6 and leptin, further demonstrating that at basal steady state the global *Zip14* KO mice models metabolic endotoxemia in humans^27^. In support of that notion, we previously showed that Zip14 expression was increased markedly in skeletal muscle of old mice which have characteristically elevated serum IL-6^28^.

ZnT2 expression is most frequently in high abundance in tissues with a secretory function including the mammary gland and exocrine pancreas^29,30^. Muscle is a secretory organ^5,31^, consequently the high expression of ZnT2 in muscle may reflect increased secretory activity. The documented secretion of proteins including IL-6, Sparc and Saa3 from muscle^5,32^ may explain in part the need for augmented ZnT2 production following LPS stimulation.

Transcript profiling of muscle of the KO and WT mice at basal steady state revealed *carbonic anhydrase*, *H19/Mir675*, *Sparc* and *Pfkfb3* were the four most up-regulated genes in GM of KO mice compared to WT mice (Table 1; Supplementary Table 1). Car3 is a muscle-specific carbonic anhydrase^33^. Sparc is a secreted muscle protein^34,35^. *Pfkfb3* is transcriptionally regulated via p38 pathway following stress and functions in glycolysis^36,37^. We propose that up-regulation of *Car3*, *Sparc* and *Pfkfb3* with *Zip14* ablation represents an adaptive response to signals related to muscle dysfunction. Of note is that Car3 may respond to oxidative stress and may be a marker of neuromuscular disease^38,39^.

Of special interest is the marked up-regulation of *H19/Mir675* in muscle of the KO mice. Postnatally, H19 expression is repressed in all tissues except skeletal muscle^14,15^. *H19* has been suggested as being anti-myogenic based on one study^40^ and pro-myogenic from another^14^. *H19* is a lncRNA that gives rise to two microRNAs (*miR-675-3p* and *miR-675-5p*) encoded within exon 1. The sequences in the microarray used here detect both *miR-675s* and those individual sequences were used in our confirmatory qPCR assays. Those assays revealed markedly greater abundance of *miR-675-5p*, but not *miR-675-3p*, in skeletal muscle of *Zip14* KO mice at steady state. miR-675-5p has been proposed to support muscle proliferation/differentiation through repression of Cdc6, a myogenic repressor in satellite cells^14,15^. In that regard it is of note that *Zip14* ablation did not influence *Cdc6* mRNA expression but did increase *TGF-*$\beta$ mRNA. TGF-$\beta$ is a known inhibitor of myogenic differentiation^41^. Therefore, considering muscle wasting is found in the *Zip14* KO mice, we conclude that *miR-675-5p* is responding as an adaptive signal to attempt stimulation of myogenesis and regeneration. Muscle wasting in the KO mice merges well with clinical findings on concurrent wasting and *miR-675* expression in humans with chronic obstructive pulmonary disease^42^. The response of both *miR-675-3p* and *miR-675-5p* show that further research is necessary to evaluate those targets of *miR-675* associated with inflammation. For example, the parent lncRNA of *miR-675*, *H19*, has been proposed as an antagonist of acute inflammation responsible for damage to intestinal epithelium^43^.

The up-regulation of *Mef2c* in muscle from the KO mice at basal steady state is also very relevant to the muscle wasting of the *Zip14* KO phenotype. Mef2c is a major transcriptional regulator of genes responsible for skeletal muscle growth and differentiation^16,21^. *Hspb7*, a gene that codes of a protein that functions in muscle atrophy and autophagy is regulated by Mef2 transcription factors^18,19^. The mechanism is through a Mef2 consensus sequence within the first 1 kb of the Hspb7 translation start site^23^. The increased Hspb7 expression we detected at the mRNA and protein levels in the *Zip14* KO mice is a likely reflection of increased transcription mediated by Mef2c activity. The increase in Mef2c expression may occur through the systemic endotoxemia and proinflammatory state of the *Zip14* KO phenotype which would include NF-$\kappa \beta$ activation. Based upon our ChIP assay we propose that NF-$\kappa \beta$ stimulates transcriptional activity of the Mef2c promoter in muscle of the *Zip14* KO mice at steady state. Since phosphorylated Mef2c (the active transcription factor) is also more abundant in the muscle of the KO mice, loss of inhibition of phosphatase activity without Zip14-mediated zinc transport may allow for sustained phosphorylation of specific signaling components. Alternatively, the increased p38 activation may lead to greater abundance of phosphorylated Mef2c. Relevant to our current studies, markedly increased *Hspb7* mRNA expression was detected in cutaneous muscle of mice showing atrophy induced by three months of microgravity during space flight^44^.

Profiling genes that are differentially induced or repressed following administration of LPS shows a markedly different pattern of expression from those at steady state. LPS administration, as a single dose, models transcriptome response that might occur in skeletal muscle after short term stress such as during acute infection or vigorous physical exercise. The novel finding that *Saa3* and *Hp* mRNAs were more up-regulated in the KO genotype after LPS suggests that Zip14 expression influences production of these acute phase proteins which appear to have specific roles in skeletal muscle during inflammation. For example, the increased *haptoglobin* (*Hp*) may limit muscle atrophy and oxidant defense^45,46^. *Saa3* is up-regulated in skeletal muscle by LPS^47^ and enhanced induction of *Saa3* in the KO mice may be a reflection of muscle degradation/remodeling^48^.

LPS administration clearly increases expression of the ubiquitin ligases *MuRF1* and *Atrogin1*, but importantly, the responses are not influenced by *Zip14* genotype. Furthermore, these genes are expressed at very low levels in mice of both genotypes when at steady state. This suggests to us that the mild metabolic endotoxemia associated with *Zip14* deletion is not sufficient to increase expression of these genes that are associated with muscle atrophy observed during severe critical illness.

It has been proposed that metastatic cancers can promote the severe, chronic muscle atrophy referred to as cachexia through a mechanism involving ZIP14^49^. *ZIP14* was up-regulated in cachectic muscle samples of humans and mice with metastatic cancer. Deletion of *Zip14* markedly reduced muscle atrophy in metastatic cancer models. The mechanism proposed was through ZIP-mediated zinc uptake which produced downregulation in expression of the myogenic transcription factors Mef2c and MyoD. It was also observed through transfection that *Zip14* represses Mef2c and MyoD expression in differentiating muscle cells. Muscle of the tumor-bearing mice used in those studies had marked elevation in expression of *MuRF1*, *Mt1* and *Mt2*. Those metabolic signatures suggest that those tumor-bearing mice were in a sustained state of proinflammatory immune activation. Furthermore, the elevated MT expression is indicative of elevated zinc trafficking in muscle of the tumor-bearing mice. In contrast, we found that *Zip14* ablation increased Mef2c expression and some genes regulated by this transcription factor, e.g. *Hspb7*. Moreover, our data suggest that during acute endotoxemia induced by LPS, Mef2c is repressed to a greater extent in muscle from *Zip14* KO mice. That reduction may have consequences as the Mef2c-regulated *Klhl31* gene was also repressed (Supplementary Tables 2 and 3). Klhl31 is associated with muscle differentiation^50^. The data of Wang *et al*. on the induction of Zip14 by TNF$\alpha$ are supportive of earlier demonstrations of Zip14 induction by IL-6 and IL-1$\beta$ in hepatocytes^11,51^ and in adipose tissue^8^. These results collectively demonstrate the influence of proinflammatory stimuli on Zip14 expression and function^52^.

Of note is that cachexia has been linked to increased expression of another Zip transporter, namely pancreatic Zip4^53^. This transporter is most widely associated with the intestinal absorption of dietary zinc^54^. The Zip4-related cachexia was attributed to increased expression of Atrogin1 and another ubiquitin E3 ligase, Ubr2. The novel mechanism proposed is through zinc-stimulated release of vesicles from the pancreas and their subsequent uptake by skeletal muscle which promotes increased Atrogin1 production^54^. These results with pancreatic Zip4 and those using metastatic cancer models^49^ with upregulation of ubiquitin ligases as a common outcome, suggest that dysfunction of both Zip4 and Zip14 may lead to cancer-induced cachexia through common mechanisms. Such conditions are not acute as genes required for cachexia occur with time in tumor bearing mice^56^. Importantly, in our experiments using a model that does not involve cancer development, the upregulation of *Atrogin1* and *MuRF1* expression following LPS in healthy mice is not dependent upon the expression of Zip14.

The involvement of Zip7 on activation of Akt signaling in cultured myoblasts has been demonstrated^55^. The mechanism proposes that Zip7-stimulated zinc release from the endoplasmic reticulum influences Akt phosphorylation and alteration of myogenic differentiation. That mechanism is in line with that which we have proposed for *Zip14* deletion using an in vivo systems approach.

Earlier, we and others reported that the *Zip14* KO mice have neurotoxic signatures related to neuronal manganese deposition and display locomotor defects^56,57^. These characterize the syndrome of manganism that resembles parkinsonism. *Zip14* KO mice exhibit a measurable reduction in locomotor activity. Hence, we cannot rule out that diminished physical activity may account for some of the abnormal muscle phenotype of the KO genotype.

**Figure 9:**
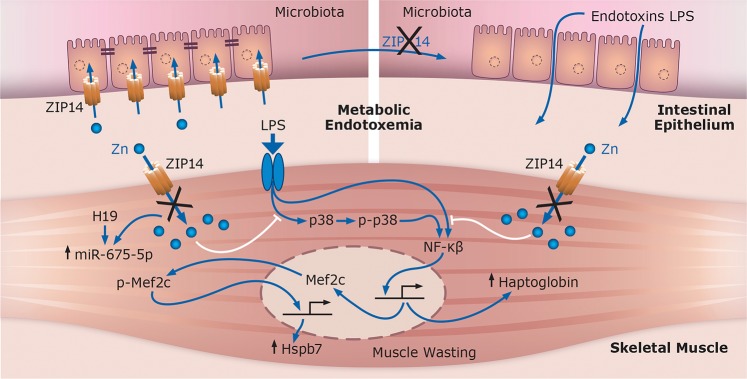
Proposed systems summary of the influence of *Zip14* ablation and metabolic endotoxemia of pathways involved in muscle wasting in mice. Global deletion of Zip14 leads to diminished intestinal barrier function and leakage of endotoxins from intestinal microbiota into the systemic circulation creating metabolic endotoxemia. Inflammation-regulated pathways activate transcription factors including NF-$\kappa \beta$ and Mef2c in skeletal muscle of the *Zip14* knockout mice creating a localized zinc deficiency leading to enhanced production of acute phase proteins and Hspb7, an autophagic protein, thus resulting in muscle wasting. White lines represent inhibitory influence of zinc ions in muscle with functioning Zip14-mediated zinc transport.

Taken together, the experiments reported here were designed to evaluate the influence of deletion of Zip14 function(s) in skeletal muscle. A proposed model to explain our data is presented in Fig. 9. Kotler has described muscle wasting as seen in the elderly, i.e., sarcopenia, as being distinct from muscle atrophy, referred to as cachexia, as associated with chronic disease such as cancer^58^. Furthermore, Frisard *et al*. have emphasized the difference between metabolic endotoxemia and critical conditions such as sepsis and cachexia^3^. Our experiments have shown aspects of both scenarios. At steady state, Zip14 ablation leads to metabolic endotoxemia, a muscle wasting phenotype, increased Hspb7, increased phosphorylated Mef2c, elevated p38 activation, increased NF-$\kappa \beta$ binding to the *Mef2c* promoter and increased *miR-675-5p* transcript abundance. Increased Hspb7, an autophagic protein^18^, may lead to wasting of muscle in the KO mice, most likely occurring through increased transcriptional activity of Mef2c produced in response to NF-$\kappa \beta$ activation. These collective metabolic imbalances limit muscle regeneration when Zip14 is deleted. In contrast, acute inflammation induced by endotoxin administration, created an acute phase response, with exaggerated activation of pathways associated with muscle atrophy, specifically those involving ubiquitin ligases, e.g. MuRF1 and Atrogin1, and markedly reduced *Mef2c* and *MyoD* expression. These acute responses are not influenced by *Zip14* ablation.

## Methods

### Mice

The *Zip14* KO and WT (C57BL/6) mice were bred at a University of Florida animal care services facility. Breeding was maintained for greater than 15 generations since 2012. Mice were given free access to commercial chow diet (Teklad 7012) and tap water, both of which were autoclaved and were maintained on a 12-hr light/dark cycle, at constant temperature and humidity. Mice used for these experiments were young adults (8–16 wks of age), with the exception of mice used for data in Fig. 3, which were 12–16 wks of age. Some mice received an intraperitoneal (i.p.) injection of LPS (2 mg/kg body weight), derived from E. coli (055:B5; Sigma-Aldrich; 5 $\times$ 104 EU/mg), or an equal amount of saline (controls) under isoflurane anesthesia. ${}^{{\rm{65}}}$Zn was administered by gavage and tissue radioactivity was measured as described^58^. *Zip14* KO and WT mice were age-matched and divided by sex and most of the mice used in this study were females. All animal procedures were approved by the University of Florida Institutional Animal Care and Use Committee and followed guidelines of the National Institutes of Health.

### Serum and muscle tissue preparation

Blood was collected from the mice by cardiac puncture under isoflurane inhalation anesthesia and serum was prepared using a two-step procedure^59^. Gastrocnemius muscle (GM) from both legs was rapidly removed from each animal and snap frozen in liquid nitrogen and stored at $-80$ ${}^{^\circ }$C. In some experiments, the soleus muscle from both legs was collected and stored as above.

### Histology of muscle tissue

Gastrocnemius muscles were excised and rapidly fixed in 10$\%$ formalin for 24 h at room temperature and then kept at 4 ${}^{^\circ }$C. Paraffin-embedded sections (5 $\mu$m) were mounted and stained with Hematoxylin and Eosin. Bright-field images of the H$\&$E stained sections were captured at 20x and 40x magnification using an EVOS (Amgmicro) microscope with a digital camera. Interstitial space of the H$\&$E stained muscle was measured using ImageJ software (National Institutes of Health and the Laboratory for Optical and Computational Instrumentation).

### Determination of metal concentrations in muscle and serum

Each GM sample was weighed and digested with concentrated nitric acid at 90 ${}^{^\circ }$C for 3h. Acid digests of muscle and serum samples were diluted with Type I lab water. Some zinc concentrations were measured by flame atomic absorption spectrophotometry (AAS). Some zinc and manganese analyses used microwave plasma-atomic emission spectrometry (MP-AES) with an Agilent 4210 instrument using emission detection at 213.857 nm for zinc and at 403.076 nm for manganese using replicate readings. Non-heme iron (NHI) was measured using the ferrozine assay^60^. Muscle phosphorus was measured by MP-AES at 214.915 nm.

### RNA isolation and quantitative real-time PCR

Total RNA was isolated from GM using TRIzol reagent (Molecular Research Center, Inc) and treated with Turbo DNA-free reagent (Ambion) to reduce DNA contamination. Total RNA concentrations were measured and assessed for purity using a NanoDrop One spectrophotometer (Thermo Fisher). Relative mRNA levels were determined by qPCR using One Step PCR Master Mix Reagents or Two Step PCR Master Mix for TaqMan analysis (Applied Biosystems) after cDNA synthesis by using High-Capacity cDNA Archive reagents (Applied Biosystems). miRNAs were reverse-transcribe using miRNA first-strand cDNA synthesis kit (QP018, Genecopoeia). Semi-quantification of *miR-675-3p* and *miR-675-5p* was performed using a miRNA q-PCR detection kit (QP016, Genecopoeia). *U6* snRNA, *TATA binding protein* and *Glyceraldehyde-3-phosphate dehydrogenase* (*Gapdh*) mRNAs were measured as normalization controls. The mature mmu-miR-675-3p (cuguaugcccuaaccgcucagu) and -5p (uggugcggaaagggcccacagu) DNA sequences were used as the forward primers, and the universal adaptor PCR primers provided in the miRNA qRT-PCR detection kit (Genecopoeia) as the reverse primer. qPCR primers unless stated here were detected with TaqMan probes.

### Protein isolation and western analysis

GM lysates were prepared in ice cold lysis buffer (20 mM Tris (pH 7.8), 137 mM NaCl, 2.7 mM KCl, 1 mM MgCl2, 1$\%$ Triton X-100, 10$\%$ (w/v) glycerol, 1 mM EDTA, 1 mM dithiothreitol)^61^ supplemented with Halt${}^{{\rm{T}}M}$ protease & phosphatase inhibitor cocktail (Thermo Scientific) using a Bullet Blender® (Next Advance) with 0.9–2.0 mm diameter stainless steel beads. The lysates were centrifuged for 20 min at 15,000 rpm and the supernatant was collected. Protein concentrations were measured using the BCA protein assay (Pierce). Proteins from each sample (40 $\mu$g) were resolved by 10$\%$ SDS-PAGE and transferred to nitrocellulose membranes. Individual blots were stained with Ponceau Red to confirm transfer efficiency. Using a molecular mass marker protein standard as a guide, blots were cut horizontally at the approximate molecular size to include the protein of interest. These strips include contiguous lanes and were incubated with the appropriate antibody. The lanes were also cut horizontally and incubated with GAPDH (range 37 kDa) and $\beta$-Tubulin (range 55 kDa) for use as the loading controls. These measures were taken to conserve antibodies, preclude the need to strip the blots and to provide the opportunity to evaluate expression of multiple proteins from the same full blot. The blots were blocked with 5$\%$ nonfat dry milk or 5$\%$ bovine serum albumin for 1h and were probed overnight with primary antibodies (usually at a concentration of 1 $\mu$g/mL). All antibodies used and their sources are presented in Supplementary Table 4. Affinity-purified Zip14 antibody was generated in house and previously characterized^11^. The membranes were incubated with secondary antibody conjugated with horseradish peroxidase (GE Healthcare). The blots were visualized with chemiluminescence ECL reagents (Thermo Fisher) and a FluorChem E Imager (ProteinSimple).

### Transcriptome profiling

Total RNA from GM was isolated with RNeasy (fibrous tissue) reagents and was DNase I treated (Qiagen). Purity and integrity were assessed as above and with an Agilent 2100 Bioanalyzer. Transcriptome profiling used the Mouse Affymetrix Clariom S Array platform (ThermoFisher). Raw hybridization data were analyzed using Affymetrix Transcriptome Analysis Console Software (version 4.0).

### Chromatin immunoprecipitation assay

Chromatin immunoprecipitation (ChIP) assays were performed with reagents (Supplementary Table 4) using specific modifications to the manufacturer’s protocol and a published procedure^62^. Each assay used 150 mg of GM that was minced and sonicated (Fisher Scientific Sonic Dismembrator 100) in 1 ml of 1$\%$ ice-cold Phosphate-buffered saline/protease inhibitor cocktails (PBS/PIC). Chromatin crosslinking was initiated by the addition of ice-cold 1$\%$ formaldehyde/PBS/PIC solution (1 ml) with mixing on a rotary shaker for 15 min at room temperature. Crosslinking was stopped by adding 100 $\mu$l of 10X glycine (0.2 M Tris, 1.5 M Glycine (pH 8.0)) and incubating on ice for 5 min. The crosslinked muscle tissue was centrifuged at 5,000 $\times$ g at 4 ${}^{^\circ }$C for 5 min and washed twice with ice-cold PBS/PIC (1 ml). After the final wash, the crosslinked muscle tissue was disaggregated by sonication (8–10 bursts $\times$ 10 seconds each) and centrifuged at 5,000 $\times$ g at 4 ${}^{^\circ }$C for 5 min. The crosslinked muscle cells were resuspended in 1ml of ChIP Cell Lysis Buffer/PIC (50 mM Hepes-KOH (pH 7), 140 mM NaCl2, 1 mM EDTA (pH 8), 10$\%$ glycerol, 0.5$\%$ NP-40 (IGEPAL), 0.25$\%$ Triton X-100, 1 mM PMSF, fresh) +5 $\mu$l 200X PIC. After resuspension, crosslinked muscle cells were incubated 5 min in ice and further homogenized by sonication (4 bursts $\times$ 10 sec each) to release the nuclei. After centrifugation at 5,000 $\times$ g at 4 ${}^{^\circ }$C for 5 min, the muscle cells were washed with 1ml of ice-cold ChIP Nuclei Wash Buffer (10 mM Hepes-KOH, pH 7, 200 mM NaCl2, 1 mM EDTA, 1 mM EGTA) +5 $\mu$l 200X PIC and centrifuged at 5,000$\times$g at 4 ${}^{^\circ }$C for 5 min. The muscle cells were resuspended and incubated for 10 min on ice with 600 $\mu$l ice-cold ChIP sonication buffer (1$\%$ SDS, 10 mM EDTA, 20 mM Tris-HCl, 150 mM NaCl, 1 mM EGTA, 0.5 mM EDTA, 0.1 mM PMSF fresh) +5 $\mu$l 200X PIC. Chromatin was fragmented by sonication using a Bioruptor® sonicator (Diagenode) with 3 runs of 10 cycles [30 sec “ON”, 30 sec “OFF”] at high power setting to generate average fragment sizes of 100–500 bp, and immunoprecipitated using anti-C/EBP$\beta$, anti-NF-$\kappa \beta$ or anti- STAT3. Isolation of immunoprecipitated chromatin and qPCR used a manufacturer’s protocol (Cell Signaling Technology). Sequences used for qPCR analysis are as follows: HP1prox-Forward, TAACACAACGCAGAGGGCCAAGTA, HP1prox-Reverse, ACGTCTCTAAGGTCACTGGCTGTT, HP1prox-Probe, GGTTTGCTTTGTGGTTTGGT. The DNA positions are denoted relative to the transcriptional start site (c/EBP$\beta$, agtatgaaGCAAgag, and STAT3, ttggttactGGAAcagcca).

### Other assays

Serum IL-6 was measured with a high sensitivity ELISA assay (BD Bioscience). Serum endotoxin levels were measured using the LAL chromogenic endotoxin quantitation system (Thermo Fisher).

### Statistical analysis

Data are presented as mean $\pm$ SEM of biological replicates. Student’s t-test was used to compare either sex or genotype differences. Multiple comparisons were conducted by analysis of variance (ANOVA) followed by Tukey post hoc test using JMP Pro13 Program (SAS version) and Prism 5 & 8 (GraphPad). P < 0.05 was considered as statistically significant. Probability in figures is indicated as *P < 0.05; **P < 0.01 and ***P < 0.001.

## Supplementary information

Supplementary Information.

Supplementary Information  2.

## Data Availability

The datasets generated during and/or analysed during the current study are available from the corresponding author on reasonable request.
