# Supplementary material for: Deletion of metal transporter Zip14 (Slc39a14) produces skeletal muscle wasting, endotoxemia, Mef2c activation and induction of miR-675 and Hspb7
Source: Sci Rep. 2020 Mar 4;10:4050. doi: 10.1038/s41598-020-61059-2 (PMC7055249; doi:10.1038/s41598-020-61059-2)
Supplement: Supplementary file 2 — Supplementary Information 2. [file 41598_2020_61059_MOESM2_ESM.pdf]

Deletion of metal transporter *Zip14* (*Slc39a14*) produces skeletal muscle wasting, endotoxemia, Mef2c activation and induction of miR-675 and Hspb7.

Jinhee Kim[1,2], Tolunay Beker Aydemir[1,3], Felix R. Jimenez-Rondan[1], Courtney H. Ruggiero[1], Min-Hyun Kim[1,4] and Robert J. Cousins[1] *

[1]Food Science and Human Nutrition Department, Center for Nutritional Sciences, College of Agricultural and Life Sciences, University of Florida, Gainesville, FL 32611; [2]Present address: Rutgers Medical School, Newark, NJ, [3]Present address: Cornell University, Ithaca, NY, [4]Present address: University of Michigan, Ann Arbor, MI. Jinhee Kim, Tolunay B. Aydemir and Felix R. Jimenez-Rondan contributed equally. Correspondence and requests for materials should be addressed to R.J.C. (email: cousins@ufl.edu).

Supplementary Figure 1.

Gastrocnemius muscle non-heme iron (NHI) and Mn and soleus muscle Zn concentrations at 18 h after LPS and Phosphorus concentrations of gastrocnemius muscle of WT and KO mice at steady state. Means ± SEM, n=3-4 mice per group.

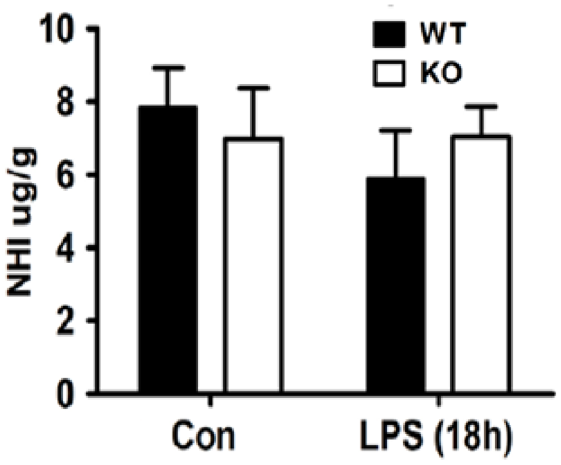

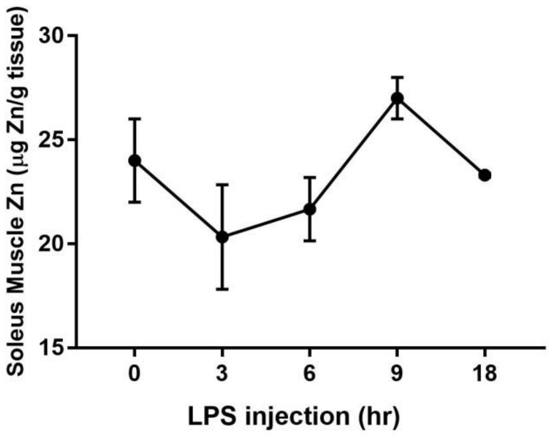

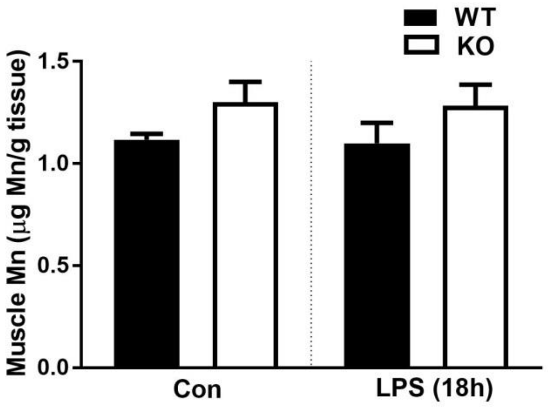

Phosphorus in Muscle at Steady State

WT = 2.74 ± 0.21 mg/g   KO = 2.54 ± 0.22 mg/g

None of these data are significant at $p<0.05$.

Supplementary Figure 2. **PAGE of gastrocnemius muscle lysate from WT and KO mice ± LPS.**

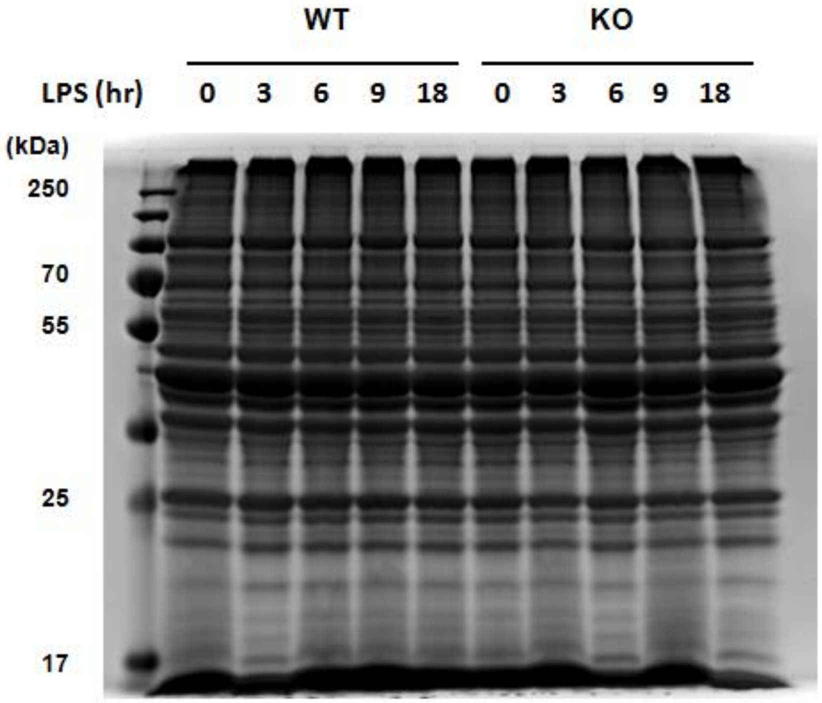

Supplementary Figure 3

Cdc6, TGF-β, and miR-675-3p mRNAs at steady state and 18 h ± LPS.

(A)

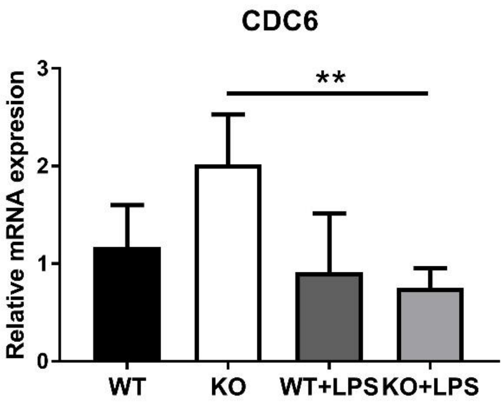

(C)

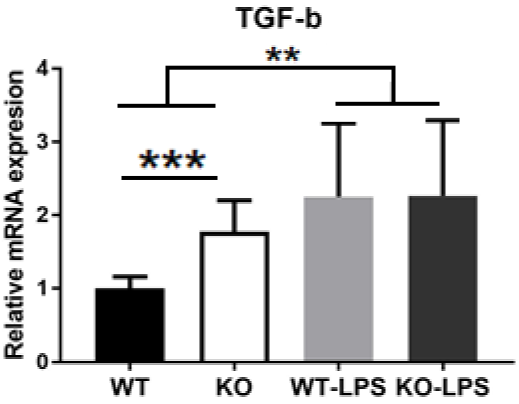

(B)

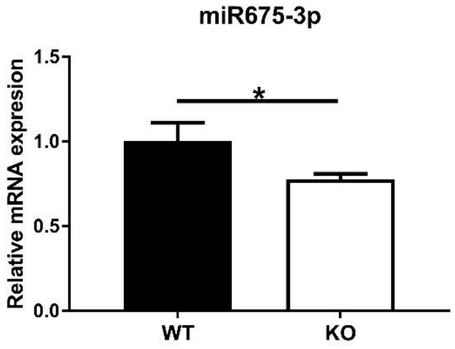

***P< 0.05; **P<0.01; ***P<0.001**

Supplementary Figure 4.

Heat map of gastrocnemius muscle RNA from WT and KO mice produced by Clariom S microarray assay.

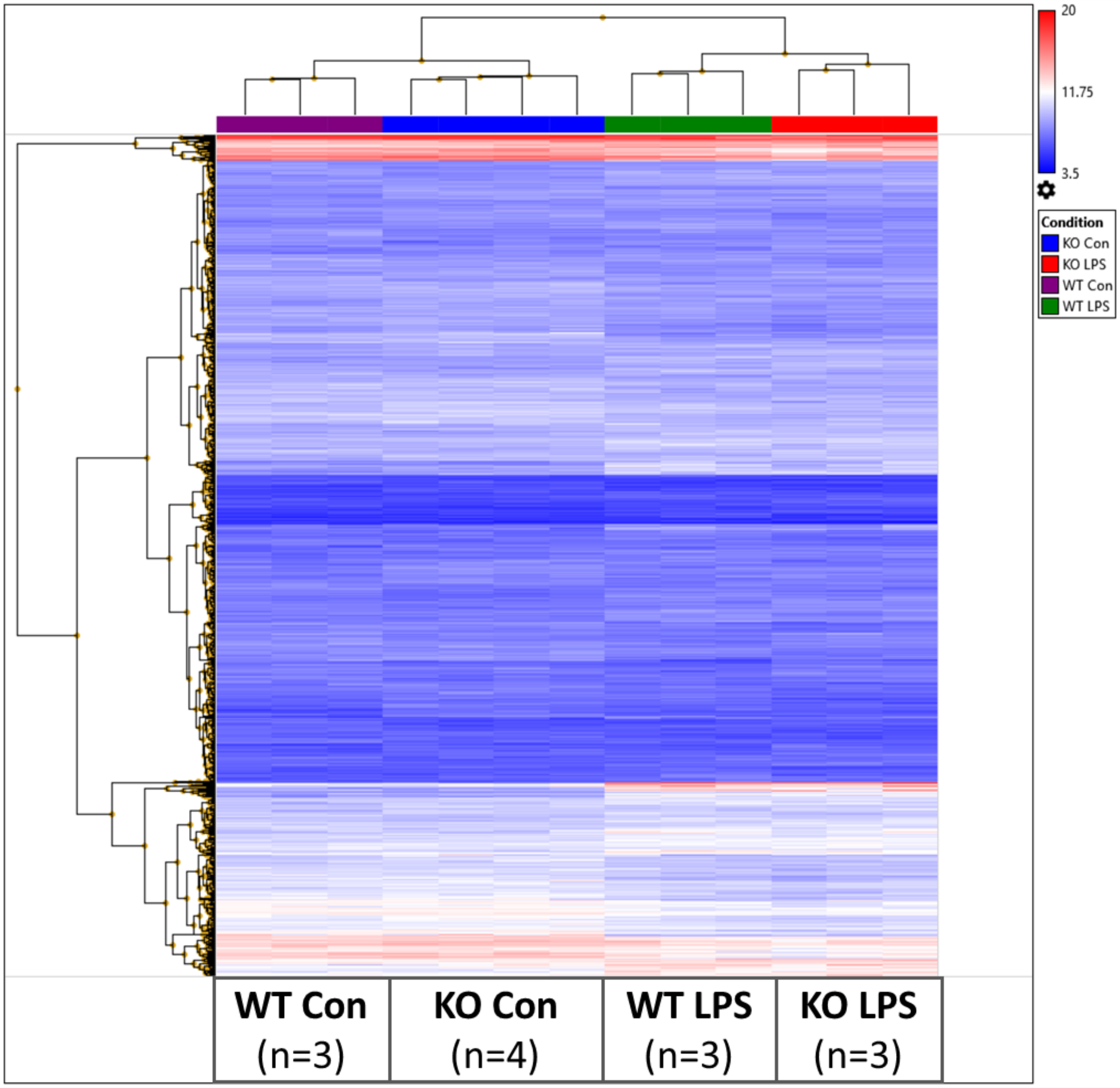

Supplementary Figure 5.

Uncropped blot for MuRF1 and atrogin1, respectively.

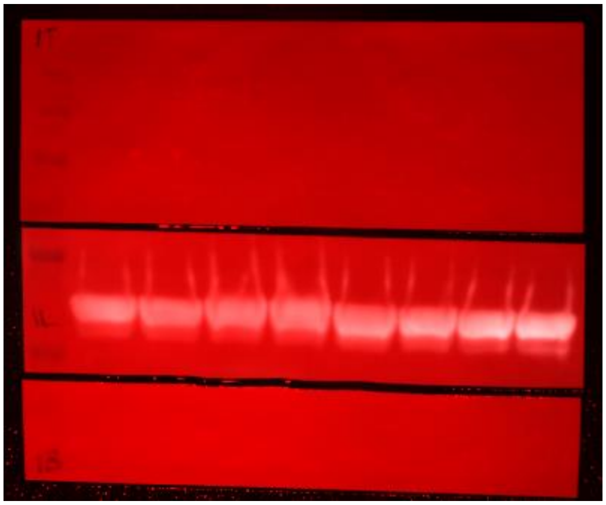

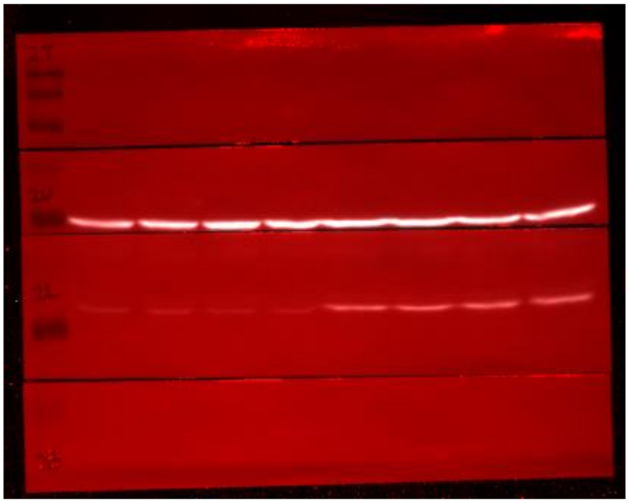

Supplementary Table 1. Selected differentially expressed genes in gastrocnemius muscle of WT vs *Zip14* KO mice at steady state.

Gene Name	Fold Change (KO/WT)	P-val
carbonic anhydrase 3	7.41	0.0001
microRNA 675; H19, imprinted maternally expressed transcript	6.63	0.0014
secreted acidic cysteine rich glycoprotein	4.41	3.69E-05
6-phosphofructo-2-kinase/fructose-2,6-biphosphatase 3	3.58	0.0002
vomeronasal 1 receptor 116	3.09	1.96E-07
heat shock protein family, member 7 (cardiovascular)	2.8	0.0154
predicted gene 10663	2.79	0.0034
decorin	2.59	0.0038
eukaryotic translation elongation factor 2	2.5	0.0012
predicted gene 13242	2.49	0.0051
glutathione peroxidase 3	2.49	0.0112
interferon inducible GTPase 1	2.47	0.0001
CD46 antigen, complement regulatory protein	2.4	0.0086
insulin-like growth factor binding protein 5	2.4	0.0331
predicted gene 10217	2.38	0.0024
alpha-kinase 3	2.35	0.0003
nebulin-related anchoring protein	2.32	0.0236
predicted gene 8906	2.3	0.0152
Map3k7 C-terminal like	2.26	0.0127
ceramide synthase 2	2.26	0.0362
pyridine nucleotide-disulphide oxidoreductase domain 1	2.25	0.001
catalase	2.25	0.0094
dopey family member 1	2.22	0.0237
vomeronasal 1 receptor 143	2.21	4.79E-05
lysosomal-associated membrane protein 2	2.21	0.0028
TP53 regulated inhibitor of apoptosis 1	2.2	0.0009
myocyte enhancer factor 2C	2.2	0.007
myosin binding protein C, fast-type	2.2	0.0148
gelsolin	2.18	0.0008
NIMA (never in mitosis gene a)-related expressed kinase 9	2.17	0.0026
predicted gene, 17571	2.17	0.0032
annexin A4	2.16	0.0208
golgi autoantigen, golgin subfamily a, 4	2.14	0.0158
muscleblind-like 1 (Drosophila)	2.13	0.0067
coatomer protein complex, subunit beta 1	2.13	0.007
sperm associated antigen 11B	2.13	0.0095
N-myc downstream regulated gene 3	2.13	0.0279
origin recognition complex, subunit 3	2.12	0.003
RIKEN cDNA 9930111J21 gene 1; RIKEN cDNA 9930111J21 gene 2	2.1	0.001

Erbb2 interacting protein	2.1	0.0128
zinc finger and BTB domain containing 20; microRNA 568	2.1	0.0165
DEAD (Asp-Glu-Ala-Asp) box polypeptide 6	2.1	0.0473
serine (or cysteine) peptidase inhibitor, clade B, member 2	2.09	0.0002
fibronectin 1	2.09	0.002
LIM domains containing 1	2.07	0.0001
zinc finger protein 318	2.07	0.0029
predicted gene 15293	2.06	0.0003
mannosidase 2, alpha 2	2.06	0.0014
homeobox containing 1	2.06	0.0045
AT rich interactive domain 1A (SWI-like); microRNA 7227	2.05	0.0012
fibromodulin	2.05	0.0079
ubiquitin specific peptidase 13 (isopeptidase T-3)	2.05	0.0152
phospholipase B domain containing 2	2.04	0.001
RIKEN cDNA 9930111J21 gene 2	2.04	0.0013
STT3, subunit of the oligosaccharyltransferase complex, homolog A (S. cerevisiae)	2.03	0.0091
actinin alpha 4	2.02	0.001
nuclear factor I/X	2.02	0.0021
fructosamine 3 kinase	2.02	0.0063
olfactory receptor 919	2.02	0.0073
olfactory receptor 310	-2.03	0.0464
olfactory receptor 1451	-2.04	0.0018
olfactory receptor 1205	-2.06	0.0378
3-hydroxy-3-methylglutaryl-Coenzyme A reductase	-2.07	0.0187
intraflagellar transport 88	-2.08	0.0229
establishment of cohesion 1 homolog 2 (S. cerevisiae)	-2.11	0.0218
adenosine deaminase, tRNA-specific 3; secretory carrier membrane protein 4	-2.15	0.0389
oocyte secreted protein 2	-2.16	0.0164
secretion regulating guanine nucleotide exchange factor	-2.18	0.0104
predicted gene 8214	-2.19	0.0295
dopamine receptor D5	-2.2	0.0005
vomeronasal 2, receptor 103	-2.26	0.0044
protein arginine N-methyltransferase 3	-2.3	0.0179
phosphatidylinositol transfer protein, cytoplasmic 1	-2.37	0.0012
RIKEN cDNA 4933408J17 gene	-2.37	0.0233
NADH dehydrogenase (ubiquinone) 1 alpha subcomplex, assembly factor 1	-2.38	0.0474
olfactory receptor 857	-2.8	0.0002

Total RNA from gastrocnemius muscle of WT (n=4) and *Zip14* KO mice was integrity-checked with a Bioanalyzer and transcript abundance was assessed using Clariom S microarrays (ThermoFisher). Data are expressed as a ratio of fold change of KO/WT and probability. Fold changes of > +2.0 or < -2.0 are shown.

Supplementary Table 2. The differentially expressed genes in gastrocnemius muscle of WT mice at 18 h ± LPS.

Gene Name	Fold Change (WT+LPS/WT)	P-val
metallothionein 1	3003.32	3.1E-18
lipocalin 2	808.3	2.5E-12
metallothionein 2	623.49	1.2E-12
tripartite motif-containing 63	206.8	2.4E-15
pyruvate dehydrogenase kinase, isoenzyme 4	140.49	3.9E-10
family with sequence similarity 134, member B	111.05	7.7E-15
arrestin domain containing 3	89.02	1.6E-12
F-box protein 32	75.58	1.7E-09
serine (or cysteine) peptidase inhibitor, clade A, member 3N	48.81	1.5E-07
cyclin-dependent kinase inhibitor 1A (P21)	41.79	2.2E-09
transmembrane protein 140	30.43	1.1E-11
cathepsin L	28	2.9E-09
protein phosphatase 1, regulatory (inhibitor) subunit 3C	22.13	2.5E-06
transforming, acidic coiled-coil containing protein 2	17.97	1.6E-06
glutamate-ammonia ligase (glutamine synthetase); microRNA 8114	17.94	2.9E-08
serum amyloid A 3	16.77	9.8E-06
patatin-like phospholipase domain containing 2	15.86	2.4E-09
ubiquitin pseudogene	15.48	1.9E-08
leucine rich repeat containing 58	14.28	4.5E-08
chemokine (C-X-C motif) ligand 13	14.22	1.3E-07
FK506 binding protein 5	12.97	3.4E-09
CCAAT/enhancer binding protein (C/EBP), delta	12.37	1.9E-09
heat shock protein family, member 7 (cardiovascular)	12.28	1.1E-06
lymphocyte antigen 6 complex, locus A	12.23	2E-07
cDNA sequence BC005537	11.74	6.1E-08
zinc finger, AN1-type domain 5	9.31	1.1E-07
ubiquitin C; ubiquitinA-52 residue ribosomal protein fusion product1	9.14	3.9E-06
solute carrier family 43, member 1	9.04	5.2E-08
myostatin	9.01	1E-06
DNA-damage inducible protein 2; regulatory solute carrier protein, family 1, member 1	8.9	1.4E-06
ubiquitin B; ubiquitin pseudogene	8.79	1.1E-08
WW domain binding protein 1 like	8.77	8.7E-08
RIKEN cDNA D230025D16 gene	8.53	5.4E-08
chemokine (C-X-C motif) ligand 9	8.27	2.6E-06
sestrin 1	8.26	5.7E-08
myogenic factor 6	8.22	2.6E-05
phosphodiesterase 4B, cAMP specific	8.17	5.1E-07
ankyrin repeat and SOCs box-containing 5	7.61	5E-07

acyl-CoA thioesterase 2	7.56	5.8E-08
TSC22 domain family, member 3	7.1	2.2E-08
activating transcription factor 4	7.08	1.7E-08
gamma-aminobutyric acid (GABA) A receptor-associated protein-like 1	7.05	5.9E-08
Erbb2 interacting protein	6.94	1.4E-06
HECT domain containing 1	6.94	0.0002
BCL2/adenovirus E1B interacting protein 3	6.88	2.7E-09
regulator of calcineurin 1	6.53	4.2E-05
predicted gene 11114 [Source:MGI Symbol;Acc:MGI:3779366]	6.42	0.027
kelch-like 38	6.39	4.7E-07
heat shock protein 1	6.12	2.4E-07
peroxiredoxin 1	6.05	1.8E-07
eukaryotic translation initiation factor 4E binding protein 1	5.86	6.9E-07
RAN binding protein 9	5.76	7.8E-07
adenosine monophosphate deaminase 3	5.75	2.6E-08
serine incorporator 3	5.65	1.5E-05
apolipoprotein D	5.64	0.0088
cathepsin D; interferon induced transmembrane protein 10	5.6	7.6E-05
musculoskeletal, embryonic nuclear protein 1	5.45	1.3E-05
molybdenum cofactor synthesis 1	5.33	6.4E-08
signal transducer and activator of transcription 3	5.25	1.8E-07
beta-2 microglobulin	5.2	0.0012
lysosomal-associated membrane protein 2	4.98	5.8E-07
acidic (leucine-rich) nuclear phosphoprotein 32 family, member A	4.93	2E-07
DnaJ (Hsp40) homolog, subfamily B, member 9	4.83	2E-07
ankyrin repeat and SOCS box-containing 11	4.78	5.4E-07
myeloid leukemia factor 1	4.75	7.3E-07
catalase	4.61	0.0004
regulator of G-protein signaling 2	4.58	2.6E-06
leucine rich repeat containing 30	4.53	1.5E-06
PDZ and LIM domain 5	4.32	5.6E-07
uncoupling protein 3 (mitochondrial, proton carrier)	4.31	1.8E-05
tubulin, alpha 1C	4.24	7.1E-06
proteasome (prosome, macropain) subunit, alpha type 7	4.23	7.6E-05
thioredoxin interacting protein	4.14	0.0014
proteasome (prosome, macropain) 26S subunit, non-ATPase, 13	4.05	0.0001
ornithine decarboxylase, structural 1	3.97	2.9E-08
microtubule-associated protein 1 light chain 3 beta	3.97	3.6E-08
proteasome (prosome, macropain) 26S subunit, ATPase, 6	3.96	6E-05
transmembrane protein 87B	3.96	0.0002
lymphocyte antigen 6 complex, locus C1	3.96	0.0008
neuroepithelial cell transforming gene 1	3.95	4.4E-06

leucine rich repeat containing 2	3.81	6.3E-08
glutamic pyruvate transaminase (alanine aminotransferase) 2	3.77	8.9E-07
tubulin, beta 4B class IVB	3.75	1.1E-05
ADP-ribosyltransferase 3	3.73	1.8E-06
protein tyrosine phosphatase 4a3	3.61	0.0003
ankyrin repeat domain 2 (stretch responsive muscle)	3.54	9.6E-05
solute carrier family 38, member 4	3.54	0.0007
protein tyrosine phosphatase 4a1; predicted gene 13363	3.53	9E-07
twinfilin, actin-binding protein, homolog 2 (Drosophila)	3.51	7.1E-07
RAR-related orphan receptor alpha	3.42	0.0026
ERBB receptor feedback inhibitor 1	3.4	0.0004
eukaryotic translation initiation factor 4A1	3.39	1.9E-06
T cell specific GTPase 2	3.39	0.0024
Kruppel-like factor 9	3.34	0.0004
serine (or cysteine) peptidase inhibitor, clade A, member 3M; serine (or cysteine) peptidase inhibitor, clade A, member 3K	3.32	6.9E-05
angiopoietin-like 7	3.29	0.0005
thioredoxin reductase 1	3.27	0.0004
outer dense fiber of sperm tails 3-like 2	3.22	5.7E-06
interferon-related developmental regulator 1	3.22	0.0002
zinc finger protein 207	3.19	1.4E-06
proteasome (prosome, macropain) 26S subunit, non-ATPase, 12	3.17	1.5E-05
proteasome (prosome, macropain) subunit, alpha type 1	3.13	5.4E-05
unc-51 like kinase 1	3.12	0.0002
haptoglobin	3.1	4.2E-06
ubiquitin specific peptidase 13 (isopeptidase T-3)	3.1	0.0035
muscle, skeletal, receptor tyrosine kinase	3.09	0.0084
RIKEN cDNA 8430408G22 gene	3.06	2.3E-05
importin 13	3.06	0.0002
interferon induced transmembrane protein 3	3.06	0.0008
DEAD (Asp-Glu-Ala-Asp) box polypeptide 21	3.05	2.6E-05
growth arrest and DNA-damage-inducible 45 gamma	3.02	1.9E-05
growth arrest and DNA-damage-inducible 45 alpha	3.01	1.9E-05
zinc finger, AN1-type domain 2A	2.98	2.4E-05
enoyl-Coenzyme A delta isomerase 2	2.98	1E-04
eukaryotic translation initiation factor 5; small nucleolar RNA, H/ACA box 28	2.97	2.5E-06
glutamate-cysteine ligase, modifier subunit	2.97	0.0219
anaphase promoting complex subunit 16	2.96	1.6E-05
zinc finger SWIM-type containing 1	2.96	0.006
thioredoxin-like 1	2.95	7.7E-05
proteasome (prosome, macropain) subunit, alpha type 3	2.95	0.001
lipin 1	2.94	4.8E-05
transmembrane protein 119	2.94	6.3E-05

sequestosome 1	2.92	4.1E-05
heterogeneous nuclear ribonucleoprotein A1; heterogeneous nuclear ribonucleoprotein A1 pseudogene	2.92	7.5E-05
nuclear protein localization 4 homolog (S. cerevisiae)	2.91	7.6E-06
proteasome (prosome, macropain) subunit, beta type 1	2.87	6.7E-06
jumonji domain containing 1C	2.86	0.0017
predicted gene, 17611 [Source:MGI Symbol;Acc:MGI:4937245]	2.86	0.0383
heat shock protein 90 alpha (cytosolic), class B member 1	2.85	9.4E-05
HERPUD family member 2	2.85	0.001
F-box protein 30	2.85	0.0046
ubiquitin A-52 residue ribosomal protein fusion product 1	2.84	1.1E-05
ribosomal protein S27, retrogene; ribosomal protein S27	2.84	0.006
perilipin 4	2.82	0.0009
connector enhancer of kinase suppressor of Ras 1	2.81	0.00008
proline-rich nuclear receptor coactivator 1	2.74	0.0001
GTPase, very large interferon inducible 1	2.74	0.0181
proteasome (prosome, macropain) 26S subunit, non-ATPase, 2	2.73	0.0001
vomeronasal 1 receptor 143	2.7	3.3E-06
POU domain, class 2, transcription factor 1	2.7	5.3E-05
B cell translocation gene 2, anti-proliferative	2.7	0.0004
predicted gene 10663 [Source:MGI Symbol;Acc:MGI:3642419]	2.67	0.0092
ferritin light polypeptide 1	2.64	2.4E-06
ribosomal protein S6	2.63	3.3E-05
nicotinamide phosphoribosyltransferase	2.61	0.0011
ribosomal protein L19	2.6	0.0203
transcription factor Dp 2	2.59	4.2E-05
motile sperm domain containing 1	2.59	6.3E-05
ubiquitin-conjugating enzyme E2G 1	2.59	0.0002
proteasome (prosome, macropain) 26S subunit, ATPase 2	2.58	1.7E-05
transport and golgi organization 2	2.58	0.0003
TSC22 domain family, member 1	2.58	0.0009
solute carrier family 43, member 3	2.55	0.0017
Shwachman-Bodian-Diamond syndrome homolog (human)	2.52	4.4E-05
coatomer protein complex, subunit beta 1	2.51	0.0026
aconitase 2, mitochondrial	2.51	0.0102
tripartite motif-containing 54	2.49	0.0024
ZW10 interactor	2.48	0.0004
ChaC, cation transport regulator 1	2.47	8.4E-06
eukaryotic translation initiation factor 4A2; small nucleolar RNA, C/D box 2; small nucleolar RNA, H/ACA box 81	2.47	0.0007
eukaryotic translation elongation factor 1 alpha 1	2.46	0.0118
cytochrome P450, family 27, subfamily a, polypeptide 1	2.45	3.2E-05
ATPase, Na+/K+ transporting, alpha 2 polypeptide	2.45	0.0006
OCIA domain containing 1	2.43	6E-05

RAD23b homolog (S. cerevisiae)	2.43	0.0005
Rab geranylgeranyl transferase, b subunit; small nucleolar RNA, C/D box 45C	2.41	4.4E-05
transmembrane protein 167	2.41	0.00006
transmembrane BAX inhibitor motif containing 1	2.4	1.7E-05
STT3, subunit of the oligosaccharyltransferase complex, homolog B (S. cerevisiae)	2.4	6.1E-05
Tax1 (human T cell leukemia virus type I) binding protein 1	2.4	0.0002
histone cluster 1, H4d	2.39	0.0003
solute carrier family 37 (glucose-6-phosphate transporter), member4	2.38	4.8E-05
annexin A4	2.38	0.004
tripartite motif-containing 27	2.37	0.0006
decorin	2.37	0.0122
proteasome (prosome, macropain) 26S subunit, non-ATPase, 11	2.35	0.0004
complement component 3	2.35	0.0133
Niemann-Pick type C1	2.34	0.0044
predicted gene 10309 [Source:MGI Symbol;Acc:MGI:3641941]	2.33	0.0001
guanylyl cyclase domain containing 1	2.33	0.0004
active BCR-related gene	2.33	0.0008
CD164 antigen	2.32	6.7E-05
poly(A) binding protein, cytoplasmic 1	2.31	0.005
myeloid cell leukemia sequence 1	2.28	4.1E-05
serine (or cysteine) peptidase inhibitor, clade B, member 6a	2.27	0.0031
zinc finger, RAN-binding domain containing 1	2.27	0.0146
transmembrane protein 176B	2.27	0.0205
centrosomal protein 85-like	2.26	0.0001
eukaryotic translation initiation factor 1	2.26	0.0005
small nuclear ribonucleoprotein polypeptide A; small nuclear ribonucleoprotein polypeptide A'	2.26	0.0333
leucine rich repeat containing 14B	2.25	0.0006
c-fos induced growth factor	2.25	0.0122
thioredoxin-like 4A	2.24	0.0294
DEAH (Asp-Glu-Ala-His) box polypeptide 15	2.23	0.0002
chaperonin containing Tcp1, subunit 2 (beta)	2.23	0.0011
cDNA sequence BC065397	2.23	0.0086
guanine nucleotide binding protein (G protein), beta polypeptide 2 like 1; small nucleolar RNA, C/D box 96A; small nucleolar RNA, C/D box 95	2.23	0.0447
ribosomal protein L13A; small nucleolar RNA, C/D box 32A; small nucleolar RNA, C/D box 33; small nucleolar RNA, C/D box 34; microRNA 5121	2.23	0.0486
2-cell-stage, variable group, member 3	2.22	0.0029
actin-binding Rho activating protein	2.22	0.0035
titin-cap	2.21	0.0031
histocompatibility 2, Q region locus 4	2.21	0.0048
transmembrane protein 50A	2.21	0.007

predicted gene, 20727	2.2	0.0014
glutathione peroxidase 5	2.2	0.0084
frizzled homolog 4 (Drosophila)	2.2	0.0149
SWI/SNF related, matrix associated, actin depenent ragulator of chromatin, subfamily a, member 5, pseudogene	2.2	0.0371
importin 7; small nucleolar RNA, H/ACA box 23	2.19	0.0024
cyclin G2	2.19	0.0042
lysyl oxidase	2.19	0.0063
ribosomal protein S7	2.19	0.0148
proline rich 30	2.19	0.0435
ribosomal protein L32	2.18	0.001
RNA binding motif protein 39	2.18	0.0141
spermidine/spermine N1-acetyl transferase 1	2.17	0.0006
arrestin domain containing 2	2.17	0.0009
ectodysplasin A2 receptor	2.17	0.0049
equatorin, sperm acrosome associated	2.17	0.0102
N-ethylmaleimide sensitive fusion protein attachment protein gamma	2.16	0.0084
spinster homolog 2	2.15	0.0004
splicing factor 1	2.14	0.0005
glutamic-oxaloacetic transaminase 1, soluble	2.14	0.0021
serine (or cysteine) peptidase inhibitor, clade A, member 3H	2.14	0.0039
RIKEN cDNA A630095E13 gene	2.14	0.0123
valosin containing protein	2.14	0.0267
CD36 antigen	2.14	0.0346
Kruppel-like factor 15	2.13	0.0009
ribosomal protein L35	2.13	0.0088
nuclear factor, interleukin 3, regulated	2.12	0.0013
UTP11-like, U3 small nucleolar ribonucleoprotein, (yeast)	2.12	0.0061
ELK4, member of ETS oncogene family	2.12	0.011
homeodomain interacting protein kinase 1	2.11	0.0006
zinc finger protein 318	2.11	0.0047
predicted gene, 21836 [Source:MGI Symbol;Acc:MGI:5434000]	2.11	0.0102
predicted gene 5458	2.11	0.0201
ATP-binding cassette, sub-family F (GCN20), member 3	2.1	0.0003
hexose-6-phosphate dehydrogenase (glucose 1-dehydrogenase)	2.1	0.0003
MAP kinase-interacting serine/threonine kinase 2	2.1	0.0004
proteasome (prosome, macropain) subunit, beta type 4	2.1	0.0015
cell division cycle 123	2.1	0.0016
leukocyte immunoglobulin-like receptor, subfamily A (with TM domain), member 6	2.1	0.0042
phosphatidylinositol 3-kinase, regulatory subunit, polypeptide 1 (p85 alpha)	2.1	0.0303
PREDICTED: predicted gene, 19404 (Gm19404), mRNA.	2.09	0.0099
WD repeat domain, phosphoinositide interacting 2	2.08	0.0009

transmembrane protein 252	2.08	0.0021
RAS related protein 1b	2.08	0.0025
UDP-glucose pyrophosphorylase 2	2.08	0.0132
kelch-like 22	2.07	0.0003
ST3 beta-galactoside alpha-2,3-sialyltransferase 5	2.07	0.001
Fanconi anemia, complementation group C	2.06	0.0067
UDP-glucose glycoprotein glucosyltransferase 1	2.05	0.001
forkhead box O1	2.05	0.0027
membrane-associated ring finger (C3HC4) 7	2.05	0.0032
RAB11B, member RAS oncogene family	2.05	0.0053
gamma-aminobutyric acid (GABA) A receptor, subunit gamma 2	2.05	0.006
vomeronasal 1 receptor 116	2.04	9.9E-05
caseinolytic mitochondrial matrix peptidase chaperone subunit	2.04	0.0005
WD repeat domain 77	2.04	0.0062
solute carrier family 40 (iron-regulated transporter), member 1	2.03	0.001
DNA-damage-inducible transcript 4	2.02	0.0004
histone cluster 1, H1c	2.02	0.0005
placenta-specific 8	2.02	0.0029
syndecan 4	2.02	0.0073
olfactory receptor 1039	2.02	0.0094
N-myc downstream regulated gene 4	2.02	0.0247
ferritin heavy polypeptide 1	2.01	6.1E-05
ADP-ribosyltransferase 1	2.01	0.0014
heterogeneous nuclear ribonucleoprotein H1	2.01	0.0027
iduronidase, alpha-L-	2.01	0.0252
vomeronasal 1 receptor 143; vomeronasal 1 receptor, 95	2	0.0015
single-stranded DNA binding protein 2	-2	0.0013
RIKEN cDNA F830016B08 gene	-2	0.0496
contactin associated protein-like 2	-2.01	0.0005
calmodulin 2	-2.01	0.0017
synaptophysin-like protein	-2.01	0.0128
EF-hand calcium binding domain 14	-2.03	0.0006
thymosin beta 15a	-2.03	0.0102
PHD finger protein 20	-2.03	0.0392
fat storage-inducing transmembrane protein 1	-2.04	0.0033
coiled-coil serine rich 2	-2.05	0.0015
ras homolog gene family, member Q	-2.06	0.0012
predicted gene 14418 [Source:MGI Symbol;Acc:MGI:3702408]	-2.06	0.027
nexilin	-2.07	0.0059
radixin	-2.07	0.0142
dermatopontin	-2.07	0.0246
ubiquitin specific peptidase 2	-2.08	0.0007
fibrinogen-like protein 1	-2.08	0.0194

NLR family, pyrin domain containing 4G	-2.09	0.0043
vomeronasal 2, receptor 72	-2.1	0.0216
growth hormone receptor	-2.11	0.0027
prolactin family 2, subfamily c, member 5	-2.12	0.0003
olfactory receptor 824	-2.12	0.0009
RIKEN cDNA 1700018C11 gene	-2.12	0.008
ankyrin repeat domain 52; microRNA 6914	-2.12	0.0124
SET and MYND domain containing 1	-2.13	0.0184
SH3-domain kinase binding protein 1	-2.15	0.0015
ST3 beta-galactoside alpha-2,3-sialyltransferase 6	-2.16	0.0008
acyl-CoA synthetase family member 2	-2.16	0.0023
transmembrane and tetratricopeptide repeat containing 1	-2.17	0.0017
DCN1, defective in cullin neddylation 1, domain containing 1 (S. cerevisiae)	-2.17	0.0048
membrane-associated ring finger (C3HC4) 9	-2.18	0.0003
muscleblind-like 1 (Drosophila)	-2.18	0.0045
ArfGAP with dual PH domains 1	-2.18	0.0113
interferon lambda receptor 1	-2.19	0.0016
olfactory receptor 357	-2.2	0.0158
predicted gene, 20738; predicted gene, 20823	-2.2	0.0402
expressed sequence AU021092	-2.21	0.0139
RAS, guanyl releasing protein 3	-2.22	0.01
uncharacterized LOC100862134 (LOC100862134), miscRNA.	-2.23	0.0008
AU RNA binding protein/enoyl-coenzyme A hydratase	-2.23	0.0033
kinesin family member 1B	-2.23	0.0061
aldo-keto reductase family 1, member C14	-2.25	0.0005
WNK lysine deficient protein kinase 1	-2.27	0.014
NADH dehydrogenase (ubiquinone) 1, subcomplex unknown, 2	-2.29	0.0046
ethanol induced 1	-2.3	0.0257
taste receptor, type 2, member 110	-2.31	0.0003
selection and upkeep of intraepithelial T cells 5	-2.31	0.0135
tripartite motif-containing 30D	-2.32	0.0193
Eph receptor A5	-2.34	0.0069
ATP synthase, H+ transporting, mitochondrial F0 complex, subunit F	-2.35	0.0088
olfactory receptor 857	-2.36	0.0065
adenosine deaminase, tRNA-specific 3; secretory carrier membrane protein 4	-2.37	0.0448
protein phosphatase 2, regulatory subunit B, alpha; protein phosphatase 2, regulatory subunit B', alpha	-2.38	0.0003
DnaJ (Hsp40) homolog, subfamily B, member 5	-2.38	0.0052
NADH dehydrogenase (ubiquinone) 1 beta subcomplex, 5	-2.38	0.0257
single-pass membrane protein with coiled-coil domains 1	-2.39	0.002
adenylosuccinate synthetase like 1	-2.4	0.0272
nuclear factor I/X	-2.43	0.0037

vomeronasal 2, receptor 16	-2.43	0.0193
transmembrane protein 64	-2.44	0.0003
oligosaccharyltransferase 4 homolog (S. cerevisiae)	-2.45	0.0005
toll-like receptor 3	-2.45	0.0275
anoctamin 5	-2.46	0.0025
predicted gene 10377; predicted gene 10375	-2.47	0.003
predicted gene 10377	-2.48	0.0058
Na+/K+ transporting ATPase interacting 1	-2.5	0.0002
transmembrane protein 233	-2.5	0.0006
vomeronasal 2, receptor 103	-2.55	0.0162
isocitrate dehydrogenase 3 (NAD+) alpha	-2.56	0.0004
protein phosphatase 2, regulatory subunit B, alpha; protein phosphatase 2, regulatory subunit B'', alpha	-2.57	0.0022
aspartate-beta-hydroxylase	-2.58	8.8E-05
NADH dehydrogenase (ubiquinone) flavoprotein 2	-2.59	0.0033
fibronectin type III and SPRY domain containing 2	-2.62	0.0005
like-glycosyltransferase	-2.62	0.029
leucine-rich repeats and transmembrane domains 1	-2.63	0.0004
ubiquitin-conjugating enzyme E2D 1	-2.64	3.8E-05
zinc finger protein 933	-2.66	0.0007
dehydrogenase/reductase (SDR family) member 7C	-2.67	1.5E-05
phosphoglucomutase 2	-2.7	0.0025
integral membrane protein 2A	-2.7	0.0033
leucine-rich single-pass membrane protein 1	-2.73	0.0003
SET domain containing (lysine methyltransferase) 8	-2.76	0.0003
hemochromatosis type 2 (juvenile) (human homolog)	-2.77	1.3E-05
quaking	-2.77	0.0002
predicted gene 14698 [Source:MGI Symbol;Acc:MGI:3709613]	-2.81	9.2E-05
D site albumin promoter binding protein	-2.81	0.0001
neogenin	-2.85	0.0002
myosin light chain kinase family, member 4	-2.87	0.0002
chloride channel, voltage-sensitive 1	-2.89	0.0002
SMEK homolog 2, suppressor of mek1 (Dictyostelium)	-2.93	0.0138
HRAS-like suppressor	-3	3E-05
olfactory receptor 1205	-3.01	0.0078
ankyrin repeat domain 23	-3.1	6.6E-05
solute carrier family 12, member 7	-3.14	0.0003
serine/threonine/tyrosine interaction protein	-3.18	0.0008
DNA-damage-inducible transcript 4-like	-3.19	0.0051
apolipoprotein B mRNA editing enzyme, catalytic polypeptide 2	-3.21	0.001
stearoyl-Coenzyme A desaturase 1	-3.27	0.0186
synaptopodin 2-like	-3.34	0.0014
protein kinase inhibitor, alpha	-3.44	0.0004

nudix (nucleoside diphosphate linked moiety X)-type motif 4	-3.48	2.5E-05
transmembrane protein 56	-3.53	0.0001
phosphatidylinositol transfer protein, cytoplasmic 1	-3.59	9E-06
F-box protein 40	-3.66	0.0003
3-hydroxyacyl-CoA dehydratase 1	-3.74	8.9E-06
calmodulin 1	-3.86	9.7E-07
SPARC-like 1	-3.89	0.0002
osteocrin	-3.93	0.0004
reticulon 4	-4.04	9.3E-05
xenotropic and polytropic retrovirus receptor 1	-4.07	3.2E-06
phosphoglycerate mutase 2	-4.22	4.8E-06
pyruvate dehyrogenase phosphatase catalytic subunit 1	-4.22	5.4E-06
phospholipase C, delta 4	-4.23	4.3E-06
ankyrin 1, erythroid	-4.34	4.9E-07
RIKEN cDNA 3425401B19 gene	-4.62	9.4E-05
insulin-like growth factor binding protein 5	-4.71	2.3E-05
calcium/calmodulin-dependent protein kinase II alpha	-4.76	0.0003
four and a half LIM domains 1	-4.88	0.0209
RIKEN cDNA 1700025G04 gene	-4.98	2.5E-06
calsequestrin 1	-5.03	1.1E-05
nuclear factor I/B	-5.56	0.0002
transducer of ErbB-2.1	-5.65	1.3E-05
S-adenosylmethionine decarboxylase 2; S-adenosylmethionine decarboxylase 1	-5.78	1.6E-06
kelch-like 31	-6.92	0.0009
myocyte enhancer factor 2C	-8.6	4.2E-08
synaptopodin 2	-10.3	2.4E-10
uncharacterized LOC102634333	-20.14	1.2E-07

Total RNA from gastrocnemius muscle of WT (n=4) and WT mice that had received LPS

18 h earlier. RNA was integrity-checked with a Bioanalyzer and transcript abundance

was assessed using Clariom S microarrays (ThermoFisher). Data are expressed as a

ratio of fold change of WT + LPS mice / WT mice and probability. Fold changes of >

+2.0 or > -2.0 are shown.

Supplementary Table 3. The differentially expressed genes in gastrocnemius Zip14 KO mice at 18 h ± LPS.

Gene Name	Fold Change (KO+LPS/KO))	P-val
metallothionein 1	2954.02	7.30E-18
lipocalin 2	587.38	6.49E-11
metallothionein 2	398.23	6.83E-12
tripartite motif-containing 63	114.05	6.38E-14
serum amyloid A 3	76.06	1.24E-06
pyruvate dehydrogenase kinase, isoenzyme 4	64.22	5.47E-08
family with sequence similarity 134, member B	56.1	7.40E-13
arrestin domain containing 3	30.25	3.95E-10
cyclin-dependent kinase inhibitor 1A (P21)	28.75	8.56E-08
transmembrane protein 140	20.24	1.56E-10
chemokine (C-X-C motif) ligand 9	18.45	2.33E-08
serine (or cysteine) peptidase inhibitor, clade A, member 3N	16.21	4.38E-06
F-box protein 32	14.68	1.71E-06
chemokine (C-X-C motif) ligand 13	13.78	7.58E-07
protein phosphatase 1, regulatory (inhibitor) subunit 3C	13.73	1.18E-06
RIKEN cDNA D230025D16 gene	12.19	1.41E-07
haptoglobin	11.99	1.21E-09
glutamate-ammonia ligase (glutamine synthetase); microRNA 8114	11.06	7.31E-06
cathepsin L	10.89	1.13E-07
activating transcription factor 4	10.04	1.33E-08
CCAAT/enhancer binding protein (C/EBP), delta	8.35	1.95E-07
TSC22 domain family, member 3	7.81	3.55E-08
musculoskeletal, embryonic nuclear protein 1	7.15	0.0002
growth arrest and DNA-damage-inducible 45 gamma	6.88	2.29E-07
FK506 binding protein 5	6.73	8.08E-07
ankyrin repeat and SOCs box-containing 5	6.49	1.47E-06
lymphocyte antigen 6 complex, locus A	6.4	8.98E-06
zinc finger, AN1-type domain 5	6.32	0.0002
leucine rich repeat containing 58	6.07	5.70E-06
peroxiredoxin 1	6.02	6.68E-07
myeloid leukemia factor 1	5.29	5.09E-07
regulator of calcineurin 1	5.16	0.0007
DnaJ (Hsp40) homolog, subfamily B, member 9	5.15	4.96E-07
proteasome (prosome, macropain) subunit, alpha type 7	5.07	0.0002
gamma-aminobutyric acid (GABA) Areceptor-assoc protein-like 1	4.85	4.71E-06
ceruloplasmin	4.65	0.0006
transforming, acidic coiled-coil containing protein 2	4.6	8.67E-05
growth arrest and DNA-damage-inducible 45 alpha	4.59	5.45E-05
serine incorporator 3	4.47	0.0007
sestrin 1	4.38	1.15E-05
ornithine decarboxylase, structural 1	4.24	2.87E-07

eukaryotic translation initiation factor 4E binding protein 1	4.24	1.35E-05
beta-2 microglobulin	4.23	0.0034
protein tyrosine phosphatase 4a1; predicted gene 13363	4.14	6.07E-06
cathepsin D; interferon induced transmembrane protein 10	4.12	0.0003
solute carrier family 43, member 1	3.98	7.25E-06
BCL2/adenovirus E1B interacting protein 3	3.82	2.31E-07
interferon-related developmental regulator 1	3.81	2.96E-05
enoyl-Coenzyme A delta isomerase 2	3.79	0.0001
patatin-like phospholipase domain containing 2	3.67	3.61E-05
syndecan 4	3.64	0.0004
predicted gene 12185; T cell specific GTPase 1	3.64	0.0021
ankyrin repeat and SOCS box-containing 11	3.61	1.46E-05
ubiquitin B; ubiquitin pseudogene	3.6	0.0001
cDNA sequence BC005537	3.53	3.69E-05
RIKEN cDNA 8430408G22 gene	3.53	0.0002
kelch-like 38	3.46	0.0006
eukaryotic translation initiation factor 4A2; small nucleolar RNA, C/D box 2; small nucleolar RNA, H/ACA box 81	3.39	0.0013
acyl-CoA thioesterase 2	3.34	9.08E-05
T cell specific GTPase 2	3.34	0.0001
neuroepithelial cell transforming gene 1	3.32	7.04E-05
proteasome (prosome, macropain) 26S subunit, non-ATPase, 13	3.32	0.0005
ubiquitin C; ubiquitin A-52 residue ribosomal protein fusion product 1	3.27	0.002
connector enhancer of kinase suppressor of Ras 1	3.22	0.0003
twinfilin, actin-binding protein, homolog 2 (Drosophila)	3.13	1.28E-06
transcription factor Dp 2	3.13	1.53E-05
lymphocyte antigen 6 complex, locus C1	3.09	0.0128
apolipoprotein D	3.08	0.0209
tubulin, beta 4B class IVB	3.04	6.48E-05
transport and golgi organization 2	3.04	0.0011
proteasome (prosome, macropain) subunit, alpha type 1	3.03	3.32E-05
anaphase promoting complex subunit 16	3.02	1.50E-05
microtubule-associated protein 1 light chain 3 beta	3.01	1.45E-06
RAR-related orphan receptor gamma	3.01	0.0003
ribosomal protein L5	3.01	0.0287
interferon induced transmembrane protein 3	3	0.0016
acyl-Coenzyme A dehydrogenase, long-chain	3	0.0097
ribosomal protein L9	2.97	0.0136
heat shock protein 1	2.96	1.80E-05
RNA binding motif protein 7	2.93	8.13E-05
serine (or cysteine) peptidase inhibitor, clade A, member 3M; serine (or cysteine) peptidase inhibitor, clade A, member 3K	2.89	5.77E-05
endothelial cell surface expressed chemotaxis and apoptosis regulator	2.88	1.07E-05
interferon induced transmembrane protein 2	2.86	0.0011
myogenic factor 6	2.85	0.0066

heat shock protein family, member 7 (cardiovascular)	2.8	0.0185
acidic (leucine-rich) nuclear phosphoprotein 32 family, member A	2.78	4.11E-05
proteasome (prosome, macropain) 26S subunit, non-ATPase, 12	2.77	0.0002
leucine rich repeat containing 30	2.77	0.0008
ribosomal protein L17	2.77	0.0464
phosphodiesterase 4B, cAMP specific	2.75	0.0003
zinc finger protein 868	2.74	0.0019
CD302 antigen	2.65	0.0016
predicted gene 10772 [Source:MGI Symbol;Acc:MGI:3704404]	2.64	0.0048
cysteine rich protein 61	2.6	0.0012
Ras-like without CAAX 1	2.59	1.68E-05
proteasome (prosome, macropain) subunit, alpha type 2	2.55	0.0015
acyl-CoA thioesterase 1	2.53	0.0002
Yip1 domain family, member 2	2.53	0.0035
protein arginine N-methyltransferase 3	2.5	0.0091
proteoglycan 4 (megakaryocyte stimulating factor, articular superficial zone protein)	2.48	1.30E-05
predicted gene 11037 [Source:MGI Symbol;Acc:MGI:3779261]	2.48	0.0281
nuclear factor, interleukin 3, regulated	2.47	0.0019
cytochrome c oxidase subunit IV isoform 1	2.47	0.0109
ubiquitin pseudogene	2.46	0.0006
zinc finger, AN1-type domain 3	2.45	0.0002
muscle, skeletal, receptor tyrosine kinase	2.43	0.0033
N-6 adenine-specific DNA methyltransferase 1 (putative)	2.42	0.0013
transmembrane BAX inhibitor motif containing 1	2.42	0.0025
adenosine monophosphate deaminase 3	2.41	0.0003
ERBB receptor feedback inhibitor 1	2.41	0.0168
PDZ and LIM domain 5	2.36	0.0007
zinc finger, DHHC domain containing 7	2.36	0.0099
adaptor protein complex AP-1, gamma 2 subunit	2.36	0.0365
nuclear protein localization 4 homolog (S. cerevisiae)	2.34	0.0004
transmembrane protein 176B	2.34	0.0008
UDP-glucose glycoprotein glucosyltransferase 2	2.34	0.0066
translocase of inner mitochondrial membrane 10	2.33	0.0006
regulator of G-protein signaling 2	2.33	0.0008
frizzled homolog 4 (Drosophila)	2.33	0.0034
lysyl oxidase	2.33	0.0227
solute carrier family 25 (mitochondrial carnitine/acylcarnitine translocase), member 20	2.31	0.0008
SRY (sex determining region Y)-box 12	2.31	0.0127
transmembrane protein 167	2.3	0.0032
intraflagellar transport 88	2.3	0.0093
Ras association (RalGDS/AF-6) domain family member 5	2.3	0.0138
uncoupling protein 3 (mitochondrial, proton carrier)	2.29	0.0055
arrestin domain containing 2	2.29	0.0081
LTV1 homolog (S. cerevisiae)	2.29	0.0306
cilia and flagella associated protein 69	2.28	0.0128

RIKEN cDNA 2900060B14 gene	2.28	0.0421
ubiquitin A-52 residue ribosomal protein fusion product 1	2.27	0.0052
ribosomal protein S15	2.27	0.0094
myostatin	2.27	0.0131
unc-51 like kinase 1	2.27	0.02
proteasome (prosome, macropain) subunit, alpha type 6	2.26	1.84E-05
ADP-ribosyltransferase 3	2.26	0.0162
regulator of cell cycle	2.25	8.25E-05
serine (or cysteine) peptidase inhibitor, clade B, member 6a	2.25	0.0031
transforming growth factor beta 1 induced transcript 1	2.24	7.06E-05
eukaryotic translation initiation factor 3, subunit M	2.23	0.0298
Shwachman-Bodian-Diamond syndrome homolog (human)	2.22	0.0002
sequestosome 1	2.22	0.0011
DnaJ (Hsp40) homolog, subfamily B, member 6	2.21	0.0011
sperm tail PG rich repeat containing 1	2.2	0.0018
predicted gene 9493 [Source:MGI Symbol;Acc:MGI:3779903]	2.2	0.0026
signal transducer and activator of transcription 3	2.2	0.005
DNA-damage-inducible transcript 4	2.2	0.0064
DnaJ (Hsp40) homolog, subfamily B, member 9 pseudogene	2.19	0.0004
molybdenum cofactor synthesis 1	2.19	0.0006
exocrine gland secreted peptide 15	2.19	0.0065
neurotensin	2.19	0.0108
proteasome (prosome, macropain) 26S subunit, non-ATPase, 4	2.18	0.0042
ribosomal protein S27, retrogene	2.17	0.0005
interferon activated gene 204	2.17	0.0025
AT rich interactive domain 5A (MRF1-like)	2.17	0.007
keratin associated protein 19-5	2.17	0.0285
ATP-binding cassette, sub-family A (ABC1), member 15	2.17	0.0295
nucleophosmin 1	2.16	0.0027
protease (prosome, macropain) 26S subunit, ATPase 5	2.15	0.0024
regulator of G-protein signaling 11	2.15	0.0137
ribosomal protein S27, retrogene; ribosomal protein S27	2.15	0.0173
interferon regulatory factor 7	2.13	0.0003
sine oculis-related homeobox 5	2.12	0.0008
zinc finger, AN1-type domain 2A	2.12	0.0014
eukaryotic translation initiation factor 1	2.12	0.0031
Ras homolog enriched in brain	2.12	0.0323
leiomodin 2 (cardiac)	2.12	0.0401
cytidine 5-triphosphate synthase; cytidine 5'-triphosphate synthase	2.11	0.0027
ribosomal protein S6	2.11	0.0043
ankyrin repeat and SOCS box-containing 3	2.11	0.0182
nephrocan	2.11	0.0207
olfactory receptor 1258	2.11	0.029
motile sperm domain containing 1	2.1	2.09E-05
WW domain binding protein 1 like	2.09	0.0066

secernin 2	2.08	0.001
proteasome (prosome, macropain) subunit, beta type 1	2.07	0.0002
outer dense fiber of sperm tails 3-like 2	2.07	0.0015
oxysterol binding protein-like 1A	2.07	0.0032
ribosomal protein S18	2.07	0.0071
solute carrier family 43, member 3	2.07	0.0131
spermine binding protein	2.07	0.0262
heat shock protein 90 alpha (cytosolic), class B member 1	2.06	0.0009
metallothionein 4	2.06	0.0024
suppressor of Ty 4A	2.06	0.0116
microtubule associated monooxygenase, calponin and LIM domain containing -like 1	2.06	0.0294
proteasome (prosome, macropain) 26S subunit, non-ATPase, 8	2.05	0.0037
lipase, member O4	2.05	0.0147
Fas (TNF receptor superfamily member 6)	2.04	0.0017
pyrin domain containing 3	2.04	0.0405
interferon (alpha and beta) receptor 2	2.03	0.0001
39S ribosomal protein L41, mitochondrial-like	2.03	0.001
EH-domain containing 1	2.03	0.0012
Kruppel-like factor 6	2.03	0.0088
proteasome (prosome, macropain) 26S subunit, ATPase, 6	2.03	0.0136
YTH domain containing 1	2.02	0.0096
histamine receptor H2	2.02	0.0118
RIKEN cDNA 4930565D16 gene	2.02	0.0124
family with sequence similarity 228, member B	2.01	0.0005
impact, RWD domain protein	2.01	0.002
CKLF-like MARVEL transmembrane domain containing 2A; CKLF-like MARVEL transmembrane domain containing 1	2.01	0.0083
phosphatidylinositol transfer protein, beta	2.01	0.0242
olfactory receptor 1077, pseudogene 1	2.01	0.0342
selection and upkeep of intraepithelial T cells 3	2	0.0033
DnaJ (Hsp40) homolog, subfamily B, member 5	-2.01	0.0007
kinesin family member 1B	-2.01	0.0007
predicted gene 11042 [Source:MGI Symbol;Acc:MGI:3779266]	-2.01	0.0018
Rho GTPase activating protein 5	-2.01	0.0026
zinc finger protein 125 [Source:MGI Symbol;Acc:MGI:1336211]	-2.01	0.0056
sorting nexin 13	-2.01	0.0092
integrin alpha FG-GAP repeat containing 1	-2.01	0.015
cysteine-rich hydrophobic domain 1	-2.01	0.0234
predicted gene 14436; predicted gene 8923	-2.01	0.0343
paired related homeobox 1	-2.02	0.0013
nuclear receptor coactivator 1	-2.02	0.0035
vomeronasal 1 receptor 129	-2.02	0.0075
RIKEN cDNA 2700060E02 gene	-2.02	0.028
DCN1, defective in cullin neddylation 1, domain containing 1 (S. cerevisiae)	-2.02	0.0292
zinc finger protein 101	-2.02	0.0391

B cell receptor associated protein 29	-2.02	0.0396
pyridine nucleotide-disulphide oxidoreductase domain 1	-2.03	0.0067
tubby like protein 4	-2.03	0.0112
signal peptide peptidase like 2A	-2.03	0.0153
vomeronasal 1 receptor 21	-2.03	0.0209
taurine upregulated gene 1	-2.03	0.038
Smg-7 homolog, nonsense mediated mRNA decay factor (C. elegans)	-2.04	0.0002
predicted gene, 21962 [Source:MGI Symbol;Acc:MGI:5439431]	-2.04	0.0009
adipogenin	-2.04	0.0074
vomeronasal 1 receptor 188	-2.04	0.0141
potassium large conductance calcium-activated channel, subfamily M, alpha member 1	-2.05	0.0193
RIKEN cDNA A630073D07 gene	-2.05	0.036
Fas-activated serine/threonine kinase	-2.06	0.001
LIM domains containing 1	-2.06	0.0014
zinc finger protein 600	-2.06	0.0031
N(alpha)-acetyltransferase 15, NatA auxiliary subunit	-2.06	0.004
acyl-CoA synthetase long-chain family member 1	-2.06	0.0078
special AT-rich sequence binding protein 1	-2.06	0.0107
etoposide induced 2.4 mRNA	-2.06	0.019
predicted gene 14325	-2.06	0.0461
eukaryotic translation initiation factor 4E	-2.07	0.0006
vascular endothelial zinc finger 1	-2.07	0.0048
olfactory receptor 1136	-2.07	0.0215
solute carrier family 44, member 2	-2.07	0.0337
kelch-like 41	-2.07	0.037
ras homolog gene family, member Q	-2.08	0.0002
EF-hand calcium binding domain 14	-2.08	0.0112
coenzyme Q5 homolog, methyltransferase (yeast)	-2.08	0.0316
major urinary protein 1	-2.08	0.0335
TBK1 binding protein 1	-2.09	0.0116
adhesion G protein-coupled receptor E1	-2.1	0.0013
angiopoietin-like 2	-2.1	0.0025
RAB1A, member RAS oncogene family	-2.1	0.0048
glutamate receptor, ionotropic, delta 2	-2.1	0.005
RAB7, member RAS oncogene family	-2.1	0.0058
exoribonuclease 3	-2.1	0.0124
adenylosuccinate synthetase like 1	-2.1	0.0219
zinc finger, BED type containing 6; zinc finger CCCH type containing 11A	-2.1	0.0391
family with sequence similarity 69, member A	-2.11	0.0004
FGGY carbohydrate kinase domain containing	-2.11	0.0012
retinoblastoma binding protein 7	-2.11	0.022
DCN1, defective in cullin neddylation 1, domain containing 2 (S. cerevisiae)	-2.12	0.0037
predicted gene 8247 [Source:MGI Symbol;Acc:MGI:3644638]	-2.12	0.004

leucine rich repeat containing 29	-2.12	0.0047
ADP-ribosylation factor guanine nucleotide-exchange factor 1(brefeldin A-inhibited)	-2.12	0.0085
transmembrane protein 233	-2.13	0.0006
N(alpha)-acetyltransferase 50, NatE catalytic subunit	-2.14	5.66E-05
serine incorporator 2	-2.14	0.0002
ST8 alpha-N-acetyl-neuraminide alpha-2,8-sialyltransferase 5	-2.14	0.0003
RAB10, member RAS oncogene family	-2.14	0.0023
olfactory receptor 196	-2.14	0.0091
hook homolog 3 (Drosophila)	-2.14	0.0453
phosphatidylinositol-3,4,5-trisphosphate-dependent Rac exchange factor 2	-2.15	0.0008
nuclear factor related to kappa B binding protein	-2.15	0.0028
actinin alpha 2	-2.16	0.0008
fatty acid synthase	-2.16	0.0061
protein phosphatase 1 (formerly 2C)-like	-2.17	0.0002
capping protein (actin filament) muscle Z-line, alpha 2	-2.17	0.0013
capping protein (actin filament) muscle Z-line, beta	-2.17	0.0018
EH domain binding protein 1-like 1	-2.17	0.0023
phospholipase A2, group VII (platelet-activating factor acetylhydrolase, plasma)	-2.17	0.005
FUN14 domain containing 1	-2.17	0.0051
aspartate-beta-hydroxylase	-2.17	0.0113
zinc finger protein 407	-2.17	0.0166
transmembrane protein 65	-2.17	0.0267
egl-9 family hypoxia-inducible factor 1	-2.18	0.0008
olfactory receptor 1215	-2.18	0.0024
myeloid leukemia factor 2	-2.18	0.0052
xin actin-binding repeat containing 2	-2.18	0.0062
neuronal regeneration related protein	-2.18	0.0066
guanine nucleotide binding protein (G protein), gamma 12	-2.18	0.0103
ankyrin repeat domain 9	-2.19	0.0101
phosphorylase kinase beta	-2.19	0.0262
signal recognition particle 54A	-2.2	0.0004
sodium channel, voltage-gated, type IV, alpha	-2.2	0.0009
solute carrier family 25 (mitochondrial carrier, citrate transporter), member 1	-2.21	0.0034
CDKN1A interacting zinc finger protein 1	-2.21	0.0095
palladin, cytoskeletal associated protein	-2.21	0.0256
dihydrodiol dehydrogenase (dimeric)	-2.21	0.0473
carboxypeptidase E	-2.22	0.0002
phosphodiesterase 4D, cAMP specific; microRNA 1904	-2.22	0.0004
folliculin interacting protein 1	-2.22	0.0005
neural precursor cell expressed, developmentally downregulated 4	-2.23	0.0001
purinergic receptor P2Y, G-protein coupled 2	-2.23	0.0005
nexilin	-2.23	0.0051
B cell CLL/lymphoma 9-like	-2.23	0.0074

heterogeneous nuclear ribonucleoprotein A2/B1	-2.23	0.0279
myeloid/lymphoid or mixed-lineage leukemia (trithorax homolog, Drosophila); translocated to, 3	-2.24	0.0014
ubiquitin-conjugating enzyme E2R 2	-2.24	0.002
cell division cycle 42	-2.24	0.003
pre-mRNA processing factor 8	-2.24	0.0296
plectin	-2.25	0.0048
solute carrier family 5 (neutral amino acid transporters, system A), member 4b	-2.25	0.0112
predicted gene 8906 [Source:MGI Symbol;Acc:MGI:3779820]	-2.25	0.0255
signal sequence receptor, gamma	-2.26	0.0001
transmembrane protein 185B	-2.26	0.0003
glycerol phosphate dehydrogenase 2, mitochondrial	-2.26	0.0005
PDZ and LIM domain 3	-2.26	0.0082
olfactory receptor 358	-2.26	0.0325
tropomodulin 4	-2.27	0.0062
collagen, type III, alpha 1	-2.27	0.0085
ubiquitin specific peptidase 47	-2.28	0.0036
La ribonucleoprotein domain family, member 4B	-2.28	0.034
AE binding protein 1	-2.29	9.90E-05
heat shock transcription factor 3	-2.29	0.0007
shisa family member 4	-2.29	0.0083
heat shock protein 8	-2.29	0.0278
dual specificity phosphatase 10	-2.3	0.0002
Cbp/p300-interacting transactivator, with Glu/Asp-rich carboxy-terminal domain, 2	-2.3	0.0032
profilin 2	-2.31	7.36E-05
Kell blood group complex subunit-related, X-linked	-2.31	0.0004
transferrin receptor	-2.31	0.0005
myosin, heavy polypeptide 7, cardiac muscle, beta	-2.31	0.0032
claudin 34C4	-2.31	0.0036
ATPase, Na+/K+ transporting, beta 2 polypeptide	-2.32	0.0055
zinc finger and BTB domain containing 44	-2.32	0.0213
calmodulin 3	-2.34	5.30E-05
dihydrolipoamide S-acetyltransferase (E2 component of pyruvate dehydrogenase complex)	-2.34	0.0007
isocitrate dehydrogenase 3 (NAD+) alpha	-2.34	0.0072
glycerol-3-phosphate acyltransferase, mitochondrial	-2.34	0.0151
collagen, type I, alpha 2	-2.35	0.0005
glutamyl-tRNA(Gln) amidotransferase, subunit C	-2.36	0.0003
ELL associated factor 2	-2.36	0.0052
avian musculoaponeurotic fibrosarcoma (v-maf) AS42 oncogene homolog	-2.37	0.0003
adhesion G protein-coupled receptor V1	-2.37	0.013
PPARGC1 and ESRR induced regulator, muscle 1	-2.38	1.07E-05
DEAD (Asp-Glu-Ala-Asp) box polypeptide 6	-2.38	0.0172
malic enzyme 1, NADP(+)-dependent, cytosolic	-2.38	0.0288
ring finger protein 10	-2.39	0.0002

mitofusin 2	-2.39	0.0036
MSS51 mitochondrial translational activator	-2.39	0.0172
selection and upkeep of intraepithelial T cells 11	-2.4	0.0112
junctophilin 1	-2.41	0.0003
nuclear factor I/C	-2.41	0.0008
protein phosphatase 2, regulatory subunit B, alpha; protein phosphatase 2, regulatory subunit B', alpha	-2.41	0.001
RNA binding motif protein 24	-2.42	0.0022
protein kinase, AMP-activated, alpha 2 catalytic subunit	-2.42	0.0124
phosphorylase kinase gamma 1	-2.43	0.0007
glutathione S-transferase, mu 1	-2.43	0.0035
synaptophysin-like protein	-2.43	0.0039
atlastin GTPase 2	-2.43	0.008
nuclear casein kinase and cyclin-dependent kinase substrate 1	-2.45	0.0033
HRAS-like suppressor	-2.46	2.50E-05
methionine adenosyltransferase II, alpha	-2.46	0.0001
zinc finger protein 106	-2.46	0.0005
malectin	-2.47	6.50E-05
predicted gene, 21798 [Source:MGI Symbol;Acc:MGI:5433962]	-2.47	0.0197
WW domain containing E3 ubiquitin protein ligase 1	-2.49	0.0013
intersectin 1 (SH3 domain protein 1A)	-2.49	0.0018
reticulon 4	-2.5	0.0031
predicted gene 10217 [Source:MGI Symbol;Acc:MGI:3642099]	-2.5	0.0048
ubiquitin specific peptidase 2	-2.51	0.0004
defensin beta 7	-2.51	0.012
BTB (POZ) domain containing 1	-2.52	4.85E-05
predicted gene, 17530	-2.52	0.0115
bone morphogenetic protein receptor, type II (serine/threonine kinase)	-2.52	0.0201
protein phosphatase 3, regulatory subunit B, alpha isoform (calcineurin B, type I)	-2.52	0.0482
solute carrier family 25 (mitochondrial carrier, Aralar), member 12	-2.53	0.0003
ring finger and CCCH-type zinc finger domains 2	-2.54	0.0006
fibromodulin	-2.55	0.004
insulin-like growth factor 2 receptor	-2.56	0.0096
predicted gene, 17374 [Source:MGI Symbol;Acc:MGI:4937008]	-2.56	0.0496
tankyrase, TRF1-interacting ankyrin-related ADP-ribose polymerase 2	-2.57	0.0002
angiomotin-like 1	-2.57	0.0005
hexokinase 2	-2.58	0.0004
protease, serine 23	-2.6	0.0004
SPARC-like 1	-2.61	0.0004
CTD nuclear envelope phosphatase 1	-2.62	0.0259
Ras-related GTP binding D	-2.63	5.81E-06
poly(rC) binding protein 2	-2.64	0.0008
predicted gene, 17571 [Source:MGI Symbol;Acc:MGI:4937205]	-2.64	0.0108
actinin alpha 4	-2.65	0.0002

neuroplastin	-2.65	0.008
peptide deformylase (mitochondrial); component of oligomeric golgi complex 8	-2.66	0.001
Map3k7 C-terminal like	-2.68	0.0003
xenotropic and polytropic retrovirus receptor 1	-2.71	4.47E-05
adenylate kinase 1	-2.71	9.32E-05
leucine-rich single-pass membrane protein 1	-2.72	0.0002
predicted gene 5725	-2.74	0.0001
predicted gene 5725; predicted gene 8720	-2.74	0.0001
predicted gene 13242	-2.74	0.0007
6-phosphofructo-2-kinase/fructose-2,6-biphosphatase 3	-2.74	0.0017
obscurin, cytoskeletal calmodulin and titin-interacting RhoGEF	-2.74	0.0024
karyopherin (importin) alpha 3	-2.74	0.0051
secreted acidic cysteine rich glycoprotein	-2.74	0.0113
kinesin family member 5B	-2.77	0.004
D site albumin promoter binding protein	-2.78	0.0005
microtubule-associated protein 4	-2.8	0.002
calcium channel, voltage-dependent, L type, alpha 1S subunit	-2.81	9.30E-05
leucine-rich repeats and transmembrane domains 1	-2.81	0.0002
transcription elongation factor A (SII), 3	-2.81	0.0053
quaking	-2.82	0.0009
pericentriolar material 1	-2.83	0.0046
T-box 15	-2.84	0.002
serine/threonine/tyrosine interaction protein	-2.84	0.0122
malate dehydrogenase 1, NAD (soluble)	-2.85	4.23E-05
AT rich interactive domain 1A (SWI-like); microRNA 7227	-2.86	5.38E-05
solute carrier family 6 (neurotransmitter transporter, creatine), member 8	-2.86	0.0001
golgi autoantigen, golgin subfamily a, 4	-2.86	0.0029
zinc fingers and homeoboxes 2	-2.86	0.0077
PHD finger protein 20	-2.88	0.0102
filamin A interacting protein 1-like	-2.89	3.62E-05
apolipoprotein B mRNA editing enzyme, catalytic polypeptide 2	-2.89	0.0004
cullin 3	-2.89	0.0017
osteoglycin	-2.92	6.44E-06
frizzled homolog 7 (Drosophila)	-2.92	7.64E-05
membrane protein, palmitoylated 6 (MAGUK p55 subfamily member 6)	-2.94	0.0178
transmembrane protein 64	-2.95	1.48E-05
ATPase, aminophospholipid transporter (APLT), class I, type 8A, member 1	-2.99	0.0072
glycerol-3-phosphate dehydrogenase 1 (soluble)	-3	7.45E-05
histidine rich calcium binding protein	-3	0.0008
LIM domain binding 3	-3.01	8.44E-05
fragile X mental retardation gene 1, autosomal homolog	-3.03	0.0019
sarcalumenin	-3.05	5.81E-05
protein inhibitor of activated STAT 2	-3.06	4.16E-05

myosin light chain kinase family, member 4	-3.06	6.74E-05
calmodulin 2	-3.06	0.0001
ATPase, Na+/K+ transporting, beta 1 polypeptide	-3.06	0.0018
ankyrin repeat domain 40	-3.08	4.05E-06
osteocrin	-3.1	0.0193
glutamine and serine rich 1	-3.12	1.54E-06
PDS5, regulator of cohesion maintenance, homolog B (S. cerevisiae)	-3.12	1.36E-05
nuclear receptor subfamily 1, group D, member 1	-3.13	4.73E-05
dystroglycan 1	-3.13	0.0083
predicted gene 11032 [Source:MGI Symbol;Acc:MGI:3779255]	-3.15	0.0002
serine/arginine repetitive matrix 2	-3.16	0.0271
muscleblind-like 2	-3.17	0.0005
myosin binding protein C, fast-type	-3.19	0.0117
myomesin 2	-3.2	0.0281
TGF-beta activated kinase 1/MAP3K7 binding protein 2	-3.21	6.72E-06
dermatopontin	-3.21	0.0009
resistin like alpha	-3.24	0.0044
neogenin	-3.27	0.0005
gelsolin	-3.28	2.88E-05
cyclin K	-3.29	7.67E-06
chloride channel, voltage-sensitive 1	-3.32	7.84E-05
ubiquitin specific peptidase 9, X chromosome	-3.33	0.0082
ankyrin repeat domain 23	-3.35	2.92E-06
heat shock protein, alpha-crystallin-related, B6	-3.36	0.0021
RIKEN cDNA 3425401B19 gene	-3.42	0.0004
nudix (nucleoside diphosphate linked moiety X)-type motif 4	-3.49	7.20E-05
transmembrane protein 56	-3.54	5.98E-05
predicted gene 4841	-3.54	0.0108
phosphodiesterase 4D interacting protein (myomegalin); microRNA 7225	-3.57	0.0075
anoctamin 5	-3.64	9.07E-05
predicted gene 10491 [Source:MGI Symbol;Acc:MGI:3642000]	-3.64	0.0078
kinesin family member 1C; microRNA 6925	-3.65	0.0004
cytoplasmic polyadenylation element binding protein 4	-3.69	5.03E-05
protein phosphatase 1, regulatory (inhibitor) subunit 3A	-3.69	0.0047
amino-terminal enhancer of split	-3.7	3.69E-05
adenosine monophosphate deaminase 1	-3.74	0.0013
NADH dehydrogenase (ubiquinone) 1 beta subcomplex, 5	-3.76	0.0129
oxysterol binding protein-like 8	-3.8	0.0002
pyruvate dehyrogenase phosphatase catalytic subunit 1	-3.81	5.05E-06
transmembrane and tetratricopeptide repeat containing 1	-3.82	3.19E-06
RIKEN cDNA F830016B08 gene	-3.83	0.0058
microRNA 675; H19, imprinted maternally expressed transcript	-3.85	0.0197
ankyrin 1, erythroid	-3.91	1.37E-05
F-box protein 40	-3.91	0.0002
hemochromatosis type 2 (juvenile) (human homolog)	-3.98	8.90E-06

carbonic anhydrase 3	-4	0.0017
homeodomain interacting protein kinase 2	-4.01	1.24E-05
keratocan	-4.02	2.22E-06
S-adenosylmethionine decarboxylase 2; S-adenosylmethionine decarboxylase 1	-4.04	0.0004
predicted gene 10663 [Source:MGI Symbol;Acc:MGI:3642419]	-4.05	0.0005
growth hormone receptor	-4.31	2.67E-05
SET and MYND domain containing 1	-4.44	2.00E-05
cardiomyopathy associated 5	-4.46	0.0012
phosphoglycerate mutase 2	-4.47	1.18E-06
cullin 5	-4.56	0.0009
phosphoglucomutase 2	-4.57	2.53E-05
calmodulin 1	-4.62	1.53E-06
phospholipase C, delta 4	-4.67	2.38E-05
alpha-kinase 3	-4.98	3.72E-06
nebulin-related anchoring protein	-5.02	0.0013
protein phosphatase 3, catalytic subunit, alpha isoform	-5.06	4.51E-06
ATP-binding cassette, sub-family C (CFTR/MRP), member 9	-5.16	0.0016
protein kinase inhibitor, alpha	-5.23	0.0003
RIKEN cDNA 1700025G04 gene	-5.41	8.89E-07
mannosidase 2, alpha 2	-5.42	2.16E-07
fibronectin type III and SPRY domain containing 2	-5.44	8.87E-06
ubiquitin-conjugating enzyme E2D 1	-5.62	1.43E-07
uracil phosphoribosyltransferase (FUR1) homolog (S. cerevisiae)	-5.67	3.97E-05
calcium/calmodulin-dependent protein kinase II alpha	-5.86	2.71E-05
nuclear factor I/B	-6.02	8.29E-06
protein phosphatase 2, regulatory subunit B, alpha; protein phosphatase 2, regulatory subunit B", alpha	-6.04	1.58E-06
ryanodine receptor 1, skeletal muscle	-6.04	5.95E-05
nebulin	-6.09	0.0194
four and a half LIM domains 1	-6.15	0.0019
muscleblind-like 1 (Drosophila)	-6.21	1.48E-06
phosphorylase kinase alpha 1	-6.4	0.0017
insulin-like growth factor binding protein 5	-6.46	3.33E-05
SET domain containing (lysine methyltransferase) 8	-6.47	1.87E-05
nuclear factor I/X	-6.54	2.20E-07
3-hydroxyacyl-CoA dehydratase 1	-6.73	4.51E-07
phosphofructokinase, muscle	-7.52	0.0006
stearoyl-Coenzyme A desaturase 1	-7.69	0.005
titin	-8.88	0.0393
WNK lysine deficient protein kinase 1	-10.86	1.21E-05
synaptopodin 2	-13.15	1.18E-10
calsequestrin 1	-14.37	1.04E-08
kelch-like 31	-18.76	1.47E-06
uncharacterized LOC102634333	-19.41	2.51E-07
myocyte enhancer factor 2C	-20.4	3.85E-09

Total RNA from gastrocnemius muscle of *Zip14* KO mice (n=4) and *Zip14* KO mice that

had received LPS 18 h earlier. RNA was integrity-checked with a Bioanalyzer and

transcript abundance was assessed using Clariom S microarrays (ThermoFisher). Data

are expressed as a ratio of fold change of KO + LPS mice / KO mice and probability.

Fold changes of > +2.0 or < -2.0 are shown.

Supplementary Table 4. Sources of specialized reagents.

Antibodies	Catalog Number	Source
Zip14		Made in house
IkBα	4814	Cell Signaling Technology
pIkB (p-Ser$^{32/36}$)	2859	Cell Signaling Technology
NF-kβ (Ser^{536}) ChIP Grade	8242	Cell Signaling Technology
p-NF-kβ (p65)	3033	Cell Signaling Technology
STAT3 ChIP Grade	4904	Cell Signaling Technology
pSTAT3 (p-Y^{705})	9145	Cell Signaling Technology
Mef2c	5030	Cell Signaling Technology
pMef2c (p-Ser^{396})	ab78888	Abcam
p38	9212	Cell Signaling Technology
p-p38 (p-Thr^{180}/Tyr^{182})	9211	Cell Signaling Technology
Hspb7	15700-1-AP	Proteintech
Gapdh	5174	Cell Signaling Technology
β-Tubulin	15115	Cell Signaling Technology
Atrogin1	AP2041	ECM Biosciences
MuRF1	55456-1-AP	Proteintech
c/EBP ChIP Grade	GTX15050	Cell Signaling Technology
ELC Anti-Rabbit IgG Horseradish peroxidase-linked whole antibody (from donkey)	NA934V	GE Healthcare
